# Correction: The association between antihypertensive treatment and serious adverse events by age and frailty: A cohort study

**DOI:** 10.1371/journal.pmed.1004885

**Published:** 2026-01-05

**Authors:** James P. Sheppard, Constantinos Koshiaris, Richard Stevens, Sarah Lay-Flurrie, Amitava Banerjee, Brandon K. Bellows, Andrew Clegg, F. D. Richard Hobbs, Rupert A. Payne, Subhashisa Swain, Juliet A. Usher-Smith, Richard J. McManus

It has come to the attention of the authors that some outcomes, related to fall events, were not included in the original analysis dataset, due to an error during data management. This has now been corrected, and the analysis re-run resulting in the corrections listed below. Furthermore, the original published manuscript described outcomes as an event associated with hospitalisation or death. However, deaths that were not preceded by a hospitalisation, were not included in the analysis. Finally, Table 1 presents the number (%) of males in the cohort, not females, as described in the original published manuscript.

In the Methods and findings subsection of the Abstract, there is an error in the fourth sentence of the paragraph. The correct sentence is: The primary outcome was hospitalisation within 10 years from falls.

In the Methods and findings subsection of the Abstract, there is an error in the 10th sentence. The correct sentence is: Antihypertensives were associated with an increased risk of hospitalisation from falls (adjusted Hazard Ratio [aHR] 1.10, 95% Confidence Interval 1.08–1.12), hypotension (aHR 1.32, 95% CI 1.29–1.35), syncope (aHR 1.20, 95% CI 1.17–1.22), AKI (aHR 1.44, 95% CI 1.41–1.47), electrolyte abnormalities (aHR 1.45, 95% CI 1.43–1.48) and primary care attendance with gout (aHR 1.35, 95% CI 1.32–1.37).

In the Methods and findings subsection of the Abstract, there is an error in the 12th sentence. The correct sentence is: In older patients (80–89 years) and those with severe frailty, this absolute risk was increased, with 38 and 39 fall events per 10,000 patients treated per year (respectively).

In the What Did the Researchers Do and Find subsection of the Author Summary, there is an error in the third and fourth bullet. The correct bullets are:

- In a total of 3,834,056 patients, blood pressure lowering treatment was associated with an increased risk of hospitalisation from falls, hypotension, syncope (but not fracture), acute kidney injury, electrolyte abnormalities and primary care consultations for gout.
- These risks were much higher in older patients and those with frailty. For example, in those aged 40–49 years 913 patients would need to be treated for 5 years to cause a serious fall. However, for those aged 80–89 years, only 53 patients would need to be treated for 5 years to cause a serious fall.

In the Outcomes subsection of the Methods, there is an error in the first, second and fourth sentences. The correct first sentence is: The primary outcome of this analysis was first hospitalisation with a primary diagnosis of a fall (defined according to ICD9 and ICD10 codes listed in S3 Table). The correct second sentence is: Secondary outcomes were first hospitalisation with a primary diagnosis of hypotension, syncope, fractures, acute kidney injury, electrolyte abnormalities and primary care attendance with gout (S3 Table and our github page for codelists). The correct fourth sentence is: Serious adverse events were defined as first hospitalisation with a primary diagnosis of any of the conditions mentioned above (with the exception of gout which was not included because it is typically less serious and usually only captured in primary care records).

In the Population Characteristics subsection of the Results, there is an error in the first sentence of the second paragraph. The correct sentence is: Patients entered the cohort throughout the period of observation (between 1998 and 2019, S7 Figure) and were followed up for a median of 7.1 years (interquartile range [IQR] 3.0 to 10.0 years).

In the Population Characteristics subsection of the Results, there is an error in the second sentence of the third paragraph. The correct sentence is: A total of 429,787 treated patients were compared to 429,787 similar controls for the matched analysis (S11 Table).

In the Primary outcome subsection of the Results, there is an error in the first, second and fifth sentences. The correct first sentence is: During follow-up, a total of 147,251 patients (3.8%) were hospitalised following a fall, including 31,386 patients (6.5%) in the exposure group and 115,865 (3.5%) in the non-exposed group. The correct second sentence is: In the primary analysis, using propensity score adjustment, antihypertensive treatment exposure was associated with an increased risk of hospitalisation from falls (adjusted Hazard Ratio [aHR] 1.10, 95% CI 1.08 to 1.12). The correct fifth sentence is: The overall absolute risk of falls with antihypertensive treatment was very low, with just six events (95% CI 5–7) per 10,000 patients treated per year, equivalent to a number needed to harm of 371 and 169 over five and 10 years respectively (Table 2).

In the Secondary outcomes subsection of the Results, there is an error in the first sentence. The correct first sentence is: Antihypertensive treatment exposure was also associated with an increased risk of hospitalisation from hypotension, syncope, acute kidney injury, electrolyte abnormalities and primary care consultations for, but not fracture (Fig 1, Table 2).

In the Subgroup and sensitivity analyses subsection of the Results, there is an error in the first and second sentences. The correct sentences are: The relative association between antihypertensive treatment and serious adverse events increased with age for acute kidney injury, electrolyte abnormalities and gout (Fig 2). These trends were less obvious in subgroups by baseline frailty (Fig 3).

In the Subgroup and sensitivity analyses subsection of the Results, there is an error in the fourth to seventh sentences. The correct sentences are: For example, the absolute risk of hospitalisation from a fall with antihypertensive treatment in individuals aged 40−49 years was 2 events (95% CI 1–3) per 10,000 patients treated per year (equivalent to a number needed to harm [NNH] of 913 at 5 years and 457 at 10 years). In those aged 80−89 years, this was increased to 38 events (95% CI 29–46) per 10,000 patients treated per year (equivalent to a NNH of 53 and 26 at 5 and 10 years respectively). Similarly, in fit patients, the absolute risk of a serious fall with antihypertensive treatment was 5 events (95% CI 4–6) per 10,000 patients treated per year (equivalent to a NNH of 414 at 5 years and 207 at 10 years). However, in those with severe frailty, this was increased to 39 events (95% CI −17–83) per 10,000 patients treated per year (equivalent to a NNH of 52 and 26 at 5 and 10 years respectively).

In the Subgroup and sensitivity analyses subsection of the Results, there is an error in the second sentence of the second paragraph. The correct sentence is: Further sensitivity analyses, using a competing risks approach to examine the primary outcome, also found no difference between the sub-hazard ratio for serious falls (adjusted Sub-Hazard Ratio 1.11, 95% CI 1.1.09 to 1.13) and the adjusted hazard ratio from the primary analysis (S13 Table).

In the Summary of main findings subsection of the Discussion, there is an error in the first sentence in the first paragraph. The correct sentence is: In this observational study of 3.8 million patients, previously untreated and with raised systolic blood pressure, antihypertensive treatment was associated with an increased risk over the subsequent decade of hospitalisation from falls, hypotension, syncope (but not fracture), acute kidney injury, electrolyte abnormalities and primary care consultations for gout.

In the Summary of main findings subsection of the Discussion, there is an error in the second sentence of the second paragraph. The correct sentence is: In older patients, the absolute risk of harm from a fall with treatment (NNH_5 _= 53) was found to be very similar to the likelihood of benefit (number needed to treat [NNT_5_] = 38)[21], and in such situations, the decision about whether or not to prescribe treatment is more finely balanced.

In the Strength and limitations subsection of the Discussion, there is an error in the third sentence of the first paragraph. The correct sentence is: Outcomes were based on secondary care data on the primary cause or hospitalisation, and therefore were less likely to be biased by any knowledge that patients were taking antihypertensive therapy.

In the Strength and limitations subsection of the Discussion, there is an error in the third sentence of the third paragraph. The correct sentence is: Similarly, the definition of acute kidney injury used here was based on clinical codes at hospital admission and maybe difference from that used in trials where kidney function is measured much more closely.

In the Implications for clinical practice subsection of the Discussion, there is an error in the third sentence of the second paragraph. The correct sentence is: By contrast, in older patients (80–90 + years), the NNH_5_ for serious falls was 37–53 (or 52 for those with severe frailty), whilst the NNT_5_ for major cardiovascular events is 38[21].

Tables 1 and 2 were uploaded incorrectly. Please see the correct Tables 1 and 2 here.

There is an error in the caption for Figs 1–3. Please see the correct Fig captions here.

Estimates from randomised controlled trials represent risk ratios rather than hazard ratios. For rare events such as the outcomes presented here, these would be expected to be equivalent. The total number of patients included in each analysis varies due exclusion of participants who experienced the outcome of interest on the index date, model convergence and variation in the matching algorithm.

The total number of patients included in each analysis varies due exclusion of participants who experienced the outcome of interest on the index date. Models adjusted for propensity score.

The total number of patients included in each analysis varies due to exclusion of participants who experienced the outcome of interest on the index date. Models adjusted for propensity score.

**Table 1:** Baseline characteristics of the study population.

Characteristic	No antihypertensive prescription during 12 month exposure period (non-exposed)		Antihypertensive prescription during 12 month exposure period (exposed)	
	Mean/number	SD/%	Mean/number	SD/%
Total population	3,349,869		484,187	
Age (years) (SD)	55.9	12.1	61.7	12.9
Sex (%male)	1,666,304	49.7%	245,498	50.7%
White ethnicity (%)*	1,477,232	67.9%	270,144	73.7%
Black ethnicity (%)*	68,806	3.2%	15,209	4.1%
South Asian ethnicity (%)*	59,856	2.8%	12,951	3.5%
Other ethnicity (%)*	569,195	26.2%	68,335	18.6%
High deprivation (IMD score of 5) (%)*†	480,976	15.4%	82,698	18.4%
Current smoking status (%)*	770,592	24.4%	98,215	21.4%
Alcohol consumption (heavy drinker) (%)*	59,238	2.4%	9,122	2.5%
Body mass index (kg/m^2^) (SD)	27.0	5.2	28.5	5.7
Systolic blood pressure (mmHg) (SD)	141.4	10.8	150.8	13.7
Diastolic blood pressure (mmHg) (SD)	83.2	9.0	88.2	11.8
QRisk2 risk score (SD)	10.8%	11.0%	22.2%	15.5%
eFrailty index score (SD)	0.04	0.05	0.08	0.06
Co-morbidities				
Stroke (%)	41,229	1.2%	19,973	4.1%
Transient ischemic attack (%)	19,309	0.6%	9,404	1.9%
Myocardial infarction (%)	18,560	0.6%	25,893	5.3%
Heart failure (%)	12,118	0.4%	13,782	2.8%
Peripheral vascular disease (%)	14,657	0.4%	7,744	1.6%
Coronary artery bypass graft (%)	3,404	0.1%	6,170	1.3%
Angina (%)	26,816	0.8%	31,356	6.5%
Atrial fibrillation (%)	34,551	1.0%	24,218	5.0%
Diabetes (%)	150,771	4.5%	70,884	14.6%
Chronic kidney disease (%)	28,219	0.8%	21,309	4.4%
Cancer (%)	116,589	3.5%	24,303	5.0%
Treatment prescriptions				
ACE inhibitors (%)	0	0%	187,209	38.7%
Angiotensin II receptor blockers (%)	0	0%	48,229	10.0%
Calcium channel blockers (%)	0	0%	141,454	29.2%
Thiazides and thiazide-like diuretics (%)	0	0%	148,652	30.7%
Beta-blockers (%)	0	0%	162,211	33.5%
Alpha-blockers (%)	0	0%	20,074	4.1%
Other antihypertensives (%)‡	0	0%	8,143	1.7%
Statins (%)	215,128	6.4%	151,603	31.3%
Antiplatelets/anticoagulants (%)	228,947	6.8%	146,849	30.3%
Anticholinergics (%)	295,101	8.8%	40,000	8.3%
Antidepressants (%)	604,344	18.0%	88,625	18.3%
Hypnotics/anxiolytics (%)	582,895	17.4%	79,284	16.4%
Opioids (%)	930,773	27.8%	140,760	29.1%

*Proportions based on the number of patients with information available (i.e., excluding those with missing values).

†IMD = indices of multiple deprivation; IMD score of 5 indicates patients in the highest quintile of deprivation (most deprived).

‡Other antihypertensives = centrally acting antihypertensives, direct renin inhibitors and vasodilators.

**Table 2:** Hazard ratios, absolute risk differences, and numbers needed to harm to cause one outcome at 5 and 10 years.

Outcome	Unadjusted analyses		Adjusted analyses*		Absolute risk difference (additional events per 10,000 patients per year)		Number needed to harm	
	Hazard ratio	95% CI	Adjusted Hazard ratio	95% CI	Events	95% CI	5 years	10 years
Falls (primary outcome)	1.93	1.91 to 1.96	1.10	1.08 to 1.12	6	5–7	371	169
Hypotension	2.43	2.39 to 2.48	1.32	1.29 to 1.35	7	6–7	434	153
Syncope	2.02	1.98 to 2.05	1.20	1.17 to 1.22	5	5–6	429	183
Fracture**	1.45	1.43 to 1.47	0.99	0.97 to 1.01	0	−1–0	–	–
Acute kidney injury	2.92	2.88 to 2.96	1.44	1.41 to 1.47	16	15–17	174	64
Electrolyte abnormalities	2.64	2.60 to 2.68	1.45	1.43 to 1.48	14	14–15	205	72
Gout	1.99	1.97 to 2.02	1.35	1.32 to 1.37	13	12–14	135	79

* Models adjusted for propensity score.

** Absolute risk difference too small to estimate number needed to harm.

**Fig 1:**
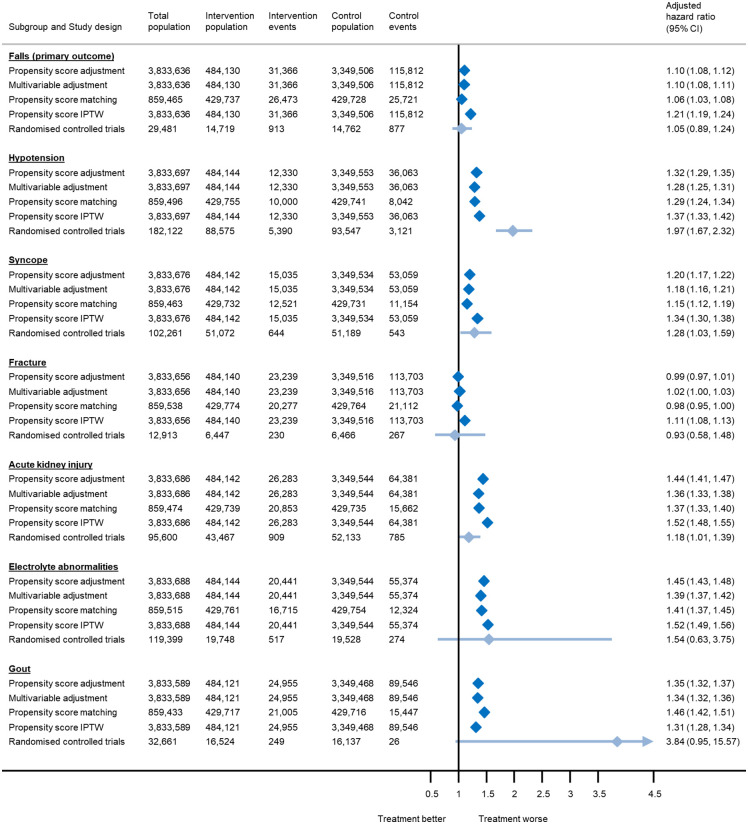
Association between antihypertensive treatment and serious adverse events leading to hospitalisation, based on analyses of electronic health records and meta-analyses of randomised controlled trials.

**Fig 2:**
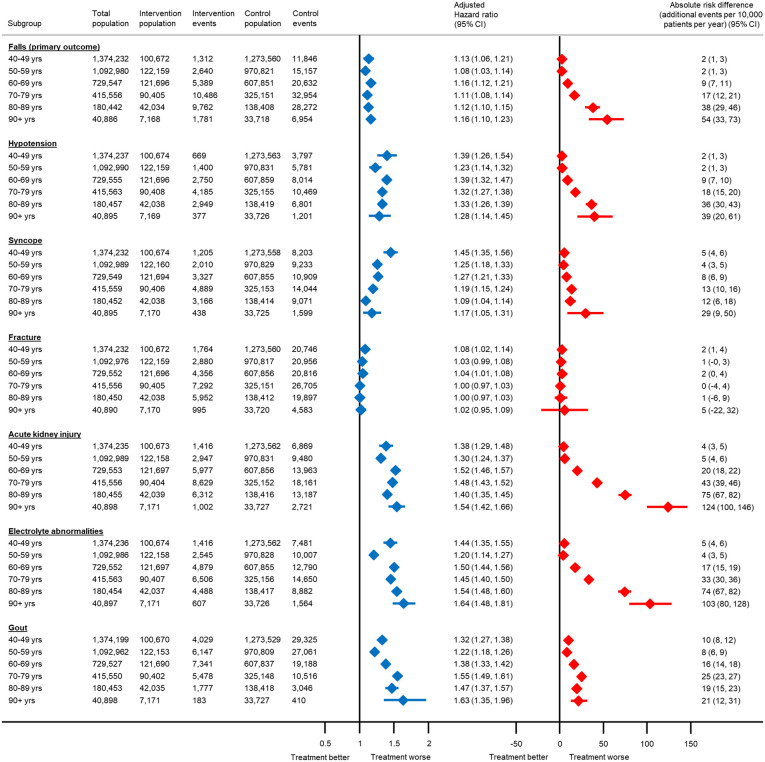
Association between antihypertensive treatment and serious adverse events leading to hospitalisation, by age at the index date.

**Fig 3:**
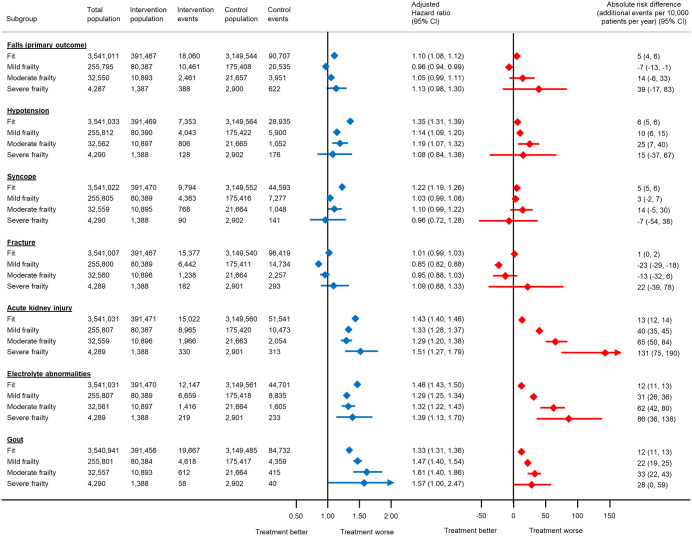
Association between antihypertensive treatment and serious adverse events leading to hospitalisation, by frailty status at the index date.
