# Supplementary material for: The association between antihypertensive treatment and serious adverse events by age and frailty: A cohort study
Source: PLoS Med. 2023 Apr 19;20(4):e1004223. doi: 10.1371/journal.pmed.1004223 (PMC10155987; doi:10.1371/journal.pmed.1004223)
Supplement: S1 Extended Methods — (DOCX) [file pmed.1004223.s001.docx]

**The association between antihypertensive treatment and serious adverse events by age and frailty: a cohort study**

**S1 Extended Methods**

*Study design and setting*

This was a retrospective observational cohort study, utilising electronic health record data from two datasets held within the Clinical Practice Research Datalink (CPRD); CPRD Gold (based on data from practices using Vision Care Electronic Health Record Software; Meddbase Software - Medical Management Systems Ltd, London, England) and CPRD Aurum (based on data from practices using Egton Medical Information Systems [EMIS] software, Leeds, England). CPRD GOLD covers over 20 million patients from 968 practices in the UK, while CPRD Aurum covers over 40 million patients from 1,491 practices. Both datasets have been shown to be representative of patients in England in terms of age, ethnicity and deprivation.[1, 2] These datasets were combined (excluding overlapping practices from the CPRD Aurum dataset) and linked at a patient level to Office for National Statistics (ONS) mortality data, basic inpatient hospital episode statistics (HES) and Index of Multiple Deprivation data (IMD). The CPRD has global ethical approval for the use of anonymised electronic health records for research purposes, subject to approval of a study protocol by their Independent Scientific Advisory Committee. The protocol for this study was given prospective approval in February 2019 (ISAC protocol number 19_042).

*Participants*

Patients were eligible if they were aged 40 years or older, registered at a linked, “up-to-standard” general practice (CPRD Gold cohort only, meaning practices achieving adequate data quality), had no previous prescription of antihypertensive therapy and had records available after the study start date (1^st^ January 1998). Eligible patients entered the cohort following their first systolic blood pressure reading ≥130 mm Hg (S2 Figure).[3, 4] Patients were excluded if they had no record of blood pressure measurement or a systolic blood pressure ≥180 mm Hg, since at this level treatment would be indicated regardless of risk of serious adverse events.[3-5] Exposure to antihypertensive medication was defined by the most recent prescriptions in the 12 months following cohort entry. The index date was defined at the end of this exposure period, after which patients were followed up for up to ten years (S2 Figure).

Patient characteristics were determined from information recorded at any point prior to the index date. Patients exited the study on the study end date (31^st^ December 2018), or when they transferred out of a registered CPRD practice, died, or experienced the specific outcome of interest.

*Outcomes*

The primary outcome of this analysis was first hospitalisation or death from a fall within 10 years of the index date (defined according to ICD9 and ICD10 codes listed in S3 Table). Secondary outcomes were first hospitalisation or death from hypotension, syncope, fractures, acute kidney injury, electrolyte abnormalities and gout (see S3 Table and <https://github.com/jamessheppard48/STRATIFY-BP/tree/Causal-inference-project> for codelists). With the exception of gout, outcomes were captured from coded hospital admissions in the Basic Inpatient Hospital Episode Statistics (primary cause of admission) and death certificates from the Office for National Statistics (primary cause of death). Gout was based on primary care coded diagnoses since patients do not typically present to hospital with this condition. In response to peer review comments, all-cause mortality and a composite outcomes of serious adverse events were examined in further post-hoc analyses. Serious adverse events were defined as first hospitalisation or death with a primary diagnosis of any of the conditions mentioned above (with the exception of gout which was not included because it is typically less serious and usually only captured in primary care records).

*Exposure*

The main exposure was prescription of any antihypertensive medication as defined in the British National Formulary:[6] Angiotensin Converting Enzyme (ACE) inhibitors, angiotensin II receptor antagonists, calcium channel blockers, thiazides and thiazide-like diuretics, beta blockers, alpha blockers, centrally acting antihypertensives, vasodilators and renin inhibitors (see S4 table). Patients were allocated to the exposure group if they were prescribed at least one antihypertensive medication during the 12 month exposure window and medications at baseline were defined by the most recent prescriptions prior to the index date. Those not exposed during this period were included in the non-exposed group. This analysis took an intention-to-treat approach, so whilst the number of participants starting therapy in the control group after the index date was estimated, such medication changes were not taken into account in the main analysis due to the complexities of combining analyses such as time-varying covariates with multiple imputation and propensity scores in analyses of very large data.

## *Covariates*

Predictors of antihypertensive treatment and the outcomes of interest were included as covariates in the analysis. These were selected based on clinical treatment guidelines,[4] previous literature[7] and expert opinion. Treatment guidelines recommend prescription on the basis of age, ethnicity, systolic/diastolic blood pressure and cardiovascular disease risk, for which we included the QRisk2 score,[8] history of cardiovascular disease (stroke, transient ischemic attack, myocardial infarction, heart failure, peripheral vascular disease, coronary artery bypass graft, angina,) and risk factors for cardiovascular disease (body mass index, total cholesterol, HDL cholesterol, smoking status and alcohol consumption, chronic kidney disease, diabetes, atrial fibrillation).[4] Inclusion of sex, indices of multiple deprivation, cancer and other prescribed medications (statins, antithrombotics) was based on a previous propensity score model predicting prescription of antihypertensive treatment.[7] Given the focus of the analysis, factors which might increase the risk of adverse events were also included, such as frailty (based on the 36 deficits specified in the validated electronic frailty index)[9] and medications associated with polypharmacy (opioids, hypnotics/anxiolytics, antidepressants and anticholinergic medications). Models were also adjusted for database from which the data were derived (Gold or Aurum) and previous history of the outcome of interest was included as an adjustment factor in the main analysis. Predictors were defined as the first occurrence of the relevant Read code before the index date, but unlike the main exposure, were not restricted to one year prior to the index date. Full code lists for variables included in this analysis can be found at <https://github.com/jamessheppard48/STRATIFY-BP/tree/Causal-inference-project>.

*Sample size*

A sample size of at least 88,380 patients (44,190 in each group) and 4,634 events was pre-specified for analyses of each outcome of interest. This assumed a clinically significant increase in the rate of each adverse event with treatment of 10%,[10] and an event rate of at least 0.5% per year in the non-exposed group, with 90% power and an alpha of 0.05. A conservative baseline event rate, lower than previously reported in the literature[11, 12] was chosen (2.2% to 7.7% per year in populations aged 55+ and 75+ years), due to the inclusion of younger patients than previously studied (40+ years).

*Statistical analysis*

The association between antihypertensive treatment and serious adverse events was examined by Cox regression using four methods to adjust for confounding: propensity score adjustment (primary analysis), multivariable adjustment, propensity score matching and inverse probability treatment weighting (using propensity score). These were expected to provide similar results and were used to demonstrate the robustness of the single method of adjustment used in subgroup analyses.

*Propensity score estimation*

Propensity scores were generated using logistic regression. Models included the covariates listed above, with continuous variables categorised to account for non-linear associations with the outcome (the use of splines/fractional polynomials was explored but led to model convergence issues). Missing data for smoking status (5.7%) and deprivation (6.7%) were low and so patients with missing values were assumed to be non-smokers and from regions with the lowest deprivation (bottom IMD quintile). Missing data for ethnicity (33.7% missing), body mass index (16.2% missing), total cholesterol (53.3% missing), HDL cholesterol (45.1% missing) and alcohol consumption (26.4% missing) were imputed using multiple imputation with chained equations, with multinomial logistic regression models employed for unordered variables (ethnicity) and ordinal logistic regression models for ordered variables. The outcome of interest from each analysis was included in separate imputation models. Analyses were undertaken with 20 imputations and model diagnostics were checked. Propensity scores were generated in each imputation dataset and combined using Rubin’s rules.[13] Propensity score model performance was assessed by the area under the receiver operating characteristic curve (AUROC) statistic, ratio of observed to expected probabilities (O/E ratio) and calibration plots. For propensity score matched analyses, treated patients were matched 1:1 to untreated patients, using the nearest neighbour method (with calliper size restricted to 0.2), and standardised mean differences were estimated pre and post matching to check the balance of baseline covariates across each group.

*Main analysis*

For the primary analysis, propensity scores were included in Cox regression models along with previous history of the outcome of interest to examine the association between antihypertensive treatment and serious adverse events. For secondary analyses, 1) Cox regression models were adjusted for the same factors included in the propensity score models, with multiple imputation used to address missing data; 2) treatment effects were compared by Cox regression in patients matched by propensity score; 3) inverse probability treatment weights were generated from the propensity score and used in a weighted Cox regression analysis with robust standard errors. The robustness of methods used to reduce confounding was examined by: 1) comparing estimates from the primary analysis to published estimates from a meta-analysis of randomised controlled trials.[14] Model assumptions were checked through inspection of Schoenfeld residuals and survival curves for the main exposure. All analyses of antihypertensive treatment effects examined the time to event for a maximum of 10 years. Absolute risk differences were estimated by comparing the baseline risk across the population assuming none were treated to the risk across the population assuming all were treated, using treatment effect estimates derived from the Cox regression models. Absolute risk differences are reported as number of events per 10,000 patients treated per year, with confidence intervals generated using bootstrap resampling (200 replications). Numbers needed to harm (NNH) were derived from the absolute risk difference at 5 and 10 years.

*Subgroup and sensitivity analyses*

Analyses of treatment associations were examined in subgroups of the population by age (grouped into ten year age bands) and frailty, determined using the electronic frailty index[9] and categorised into fit (score = 0-0.12), mild (score = >0.12-0.24), moderate (score = >0.24-0.36) and severe frailty (score = >0.36).

Sensitivity analyses were undertaken to test the assumptions made to deal with missing smoking and deprivation data, including them in the imputation models described previously, and using the missing indicator method. Further sensitivity analyses examined the impact of competing risks on the treatment effect estimates, where the association between antihypertensives and falls (the primary outcome) was examined using a Fine-Gray competing risks model, with death from any cause (apart from falls) treated as a competing risk.

Our initial protocol included a planned analysis of positive and negative controls to examine the robustness of methods used to reduce confounding. However, we were unable to define satisfactory positive or negative controls. As a result, this analysis was not included, since whatever the findings, it would not be able to sufficiently prove the presence or absence of confounding in our main analysis.

*References*

1. Herrett E, Gallagher AM, Bhaskaran K, Forbes H, Mathur R, van Staa T, et al. Data Resource Profile: Clinical Practice Research Datalink (CPRD). International journal of epidemiology. 2015;44(3):827-36. Epub 2015/06/08. doi: 10.1093/ije/dyv098. PubMed PMID: 26050254; PubMed Central PMCID: PMCPMC4521131.

2. Wolf A, Dedman D, Campbell J, Booth H, Lunn D, Chapman J, et al. Data resource profile: Clinical Practice Research Datalink (CPRD) Aurum. International journal of epidemiology. 2019;48(6):1740-g. Epub 2019/03/13. doi: 10.1093/ije/dyz034. PubMed PMID: 30859197; PubMed Central PMCID: PMCPMC6929522.

3. Whelton PK, Carey RM, Aronow WS, Casey DE, Jr., Collins KJ, Dennison Himmelfarb C, et al. 2017 ACC/AHA/AAPA/ABC/ACPM/AGS/APhA/ASH/ASPC/NMA/PCNA Guideline for the Prevention, Detection, Evaluation, and Management of High Blood Pressure in Adults: A Report of the American College of Cardiology/American Heart Association Task Force on Clinical Practice Guidelines. Hypertension (Dallas, Tex : 1979). 2018;71(6):e13-e115. Epub 2017/11/15. doi: 10.1161/hyp.0000000000000065. PubMed PMID: 29133356.

4. Williams B, Mancia G, Spiering W, Agabiti Rosei E, Azizi M, Burnier M, et al. 2018 ESC/ESH Guidelines for the management of arterial hypertension. European heart journal. 2018;39(33):3021-104. Epub 2018/08/31. doi: 10.1093/eurheartj/ehy339. PubMed PMID: 30165516.

5. National Guideline Centre. National Institute for Health and Care Excellence. Hypertension in adults: diagnosis and management [NICE guideline 136]. London: Royal College of Physicians (UK); 2019.

6. Royal Pharmaceutical Society. British National Formulary <https://bnf.nice.org.uk/>: BMJ Group andPharmaceutical Press 2022 [02/02/2022].

7. Sheppard JP, Stevens S, Stevens R, Martin U, Mant J, Hobbs FDR, et al. Benefits and Harms of Antihypertensive Treatment in Low-Risk Patients With Mild Hypertension. JAMA internal medicine. 2018;178(12):1626-34. Epub 2018/11/02. doi: 10.1001/jamainternmed.2018.4684. PubMed PMID: 30383082.

8. Hippisley-Cox J, Coupland C, Vinogradova Y, Robson J, Minhas R, Sheikh A, et al. Predicting cardiovascular risk in England and Wales: prospective derivation and validation of QRISK2. BMJ (Clinical research ed). 2008;336(7659):1475-82. Epub 2008/06/25. doi: 10.1136/bmj.39609.449676.25. PubMed PMID: 18573856; PubMed Central PMCID: PMCPmc2440904.

9. Clegg A, Bates C, Young J, Ryan R, Nichols L, Ann Teale E, et al. Development and validation of an electronic frailty index using routine primary care electronic health record data. Age and ageing. 2016;45(3):353-60. Epub 2016/03/06. doi: 10.1093/ageing/afw039. PubMed PMID: 26944937; PubMed Central PMCID: PMCPMC4846793.

10. Gribbin J, Hubbard R, Gladman JR, Smith C, Lewis S. Risk of falls associated with antihypertensive medication: population-based case-control study. Age and ageing. 2010;39(5):592-7. Epub 2010/07/24. doi: 10.1093/ageing/afq092. PubMed PMID: 20650874.

11. Stalenhoef PA, Diederiks JP, de Witte LP, Schiricke KH, Crebolder HF. Impact of gait problems and falls on functioning in independent living persons of 55 years and over: a community survey. Patient education and counseling. 1999;36(1):23-31. Epub 1999/02/26. PubMed PMID: 10036557.

12. Tinetti ME, Speechley M, Ginter SF. Risk factors for falls among elderly persons living in the community. The New England journal of medicine. 1988;319(26):1701-7. Epub 1988/12/29. doi: 10.1056/nejm198812293192604. PubMed PMID: 3205267.

13. Rubin DB. Multiple Imputation for Nonresponse in Surveys. New York: John Wiley & Sons; 1987.

14. Albasri A, Hattle M, Koshiaris C, Dunnigan A, Paxton B, Fox SE, et al. Association between antihypertensive treatment and adverse events: systematic review and meta-analysis. BMJ (Clinical research ed). 2021;372:n189. doi: 10.1136/bmj.n189.
