# Supplementary material for: The association between antihypertensive treatment and serious adverse events by age and frailty: A cohort study
Source: PLoS Med. 2023 Apr 19;20(4):e1004223. doi: 10.1371/journal.pmed.1004223 (PMC10155987; doi:10.1371/journal.pmed.1004223)
Supplement: S1 Fig — Patients were eligible at cohort entry if they were aged 40 years or older, registered at a linked, “up-to-standard” general practice, had records available after the study start date (1 January 1998), had no previous prescription of antihypertensive therapy and a single systolic blood pressure reading between 130–179 mm Hg. (DOCX) [file pmed.1004223.s002.docx]

**S1 Figure.** Definition of time periods used to define the cohort and follow-up periods.


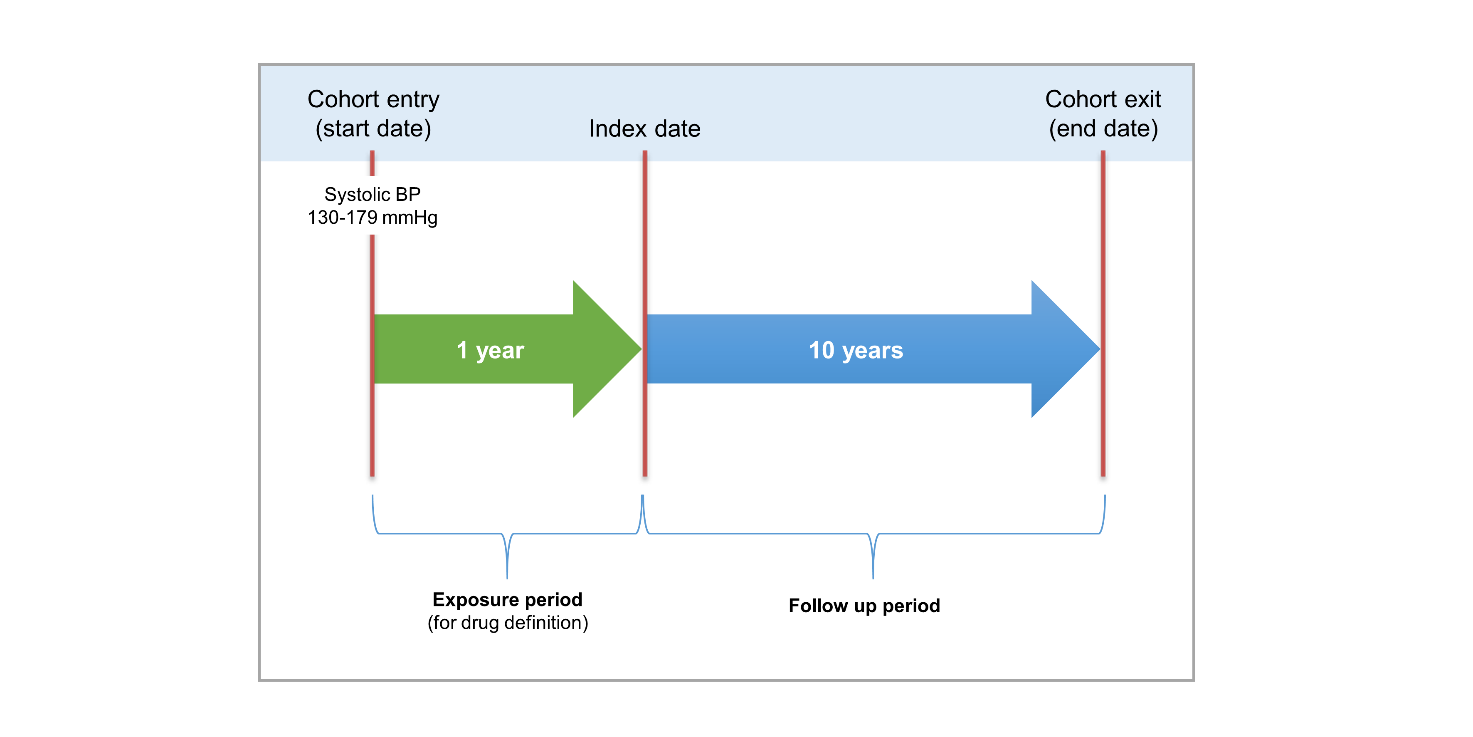


Patients were eligible at cohort entry if they were aged 40 years or older, registered at a linked, “up-to-standard” general practice, had records available after the study start date (1st January 1998), had no previous prescription of antihypertensive therapy and a single systolic blood pressure reading between 130-179 mm Hg
