# Supplementary material for: The association between antihypertensive treatment and serious adverse events by age and frailty: A cohort study
Source: PLoS Med. 2023 Apr 19;20(4):e1004223. doi: 10.1371/journal.pmed.1004223 (PMC10155987; doi:10.1371/journal.pmed.1004223)
Supplement: S2 Fig — CPRD, Clinical Practice Research Datalink; mm Hg, millimetres of mercury. (DOCX) [file pmed.1004223.s003.docx]

**S2 Figure.** Flow diagram showing selection of patient records for inclusion in the study


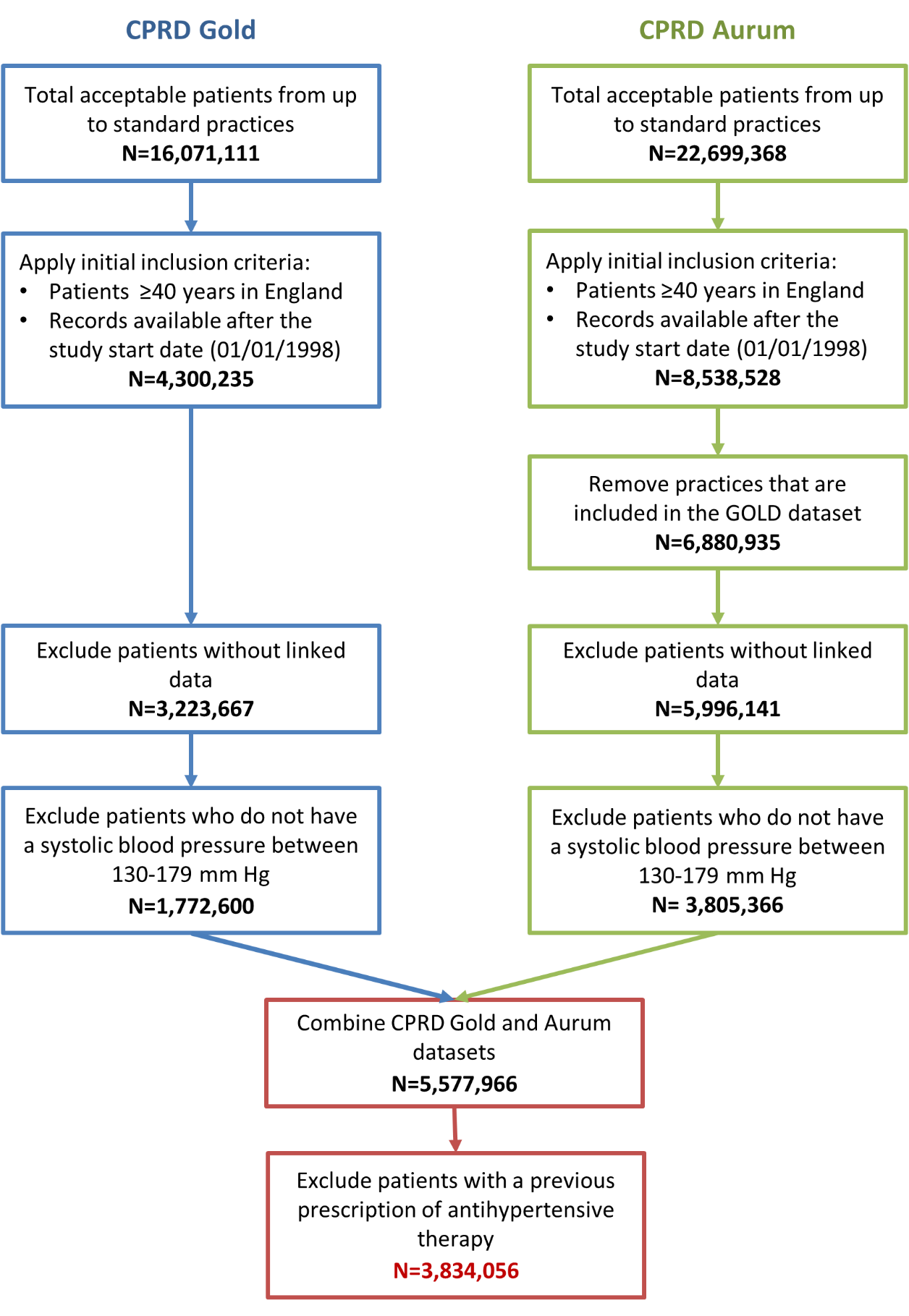


CPRD = Clinical Practice Research Datalink; mm Hg = Millimetres of mercury
