# Supplementary material for: The association between antihypertensive treatment and serious adverse events by age and frailty: A cohort study
Source: PLoS Med. 2023 Apr 19;20(4):e1004223. doi: 10.1371/journal.pmed.1004223 (PMC10155987; doi:10.1371/journal.pmed.1004223)
Supplement: S3 Fig — (DOCX) [file pmed.1004223.s004.docx]

**S3 Figure.** Percentage of patients with an index date in each year of the observational period

3%

01/01/2000

01/01/2005

01/01/2010

01/01/2015

01/01/2020

Index date

1%

2%

0%
