# Supplementary material for: The association between antihypertensive treatment and serious adverse events by age and frailty: A cohort study
Source: PLoS Med. 2023 Apr 19;20(4):e1004223. doi: 10.1371/journal.pmed.1004223 (PMC10155987; doi:10.1371/journal.pmed.1004223)
Supplement: S4 Fig — AUC, area under the curve; CITL, calibration in the large; E:O, expected over observed ratio. (DOCX) [file pmed.1004223.s005.docx]

**S4 Figure.** Propensity score model performance

E:O = Expected over observed ratio; CITL = Calibration in the large; AUC = Area under the curve
