# Supplementary material for: The association between antihypertensive treatment and serious adverse events by age and frailty: A cohort study
Source: PLoS Med. 2023 Apr 19;20(4):e1004223. doi: 10.1371/journal.pmed.1004223 (PMC10155987; doi:10.1371/journal.pmed.1004223)
Supplement: S1 Table — (DOCX) [file pmed.1004223.s006.docx]

**S1 Table.** Codes used to define serious adverse event outcomes

| **Outcome** | **ICD9/10 code** | **Description** |
| --- | --- | --- |
| **Falls (primary outcome)** | W01 | Fall on same level from slipping, tripping & stumbling |
|  | W05 | Fall involving wheelchair |
|  | W06 | Fall involving bed |
|  | W07 | Fall involving chair |
|  | W08 | Fall involving other furniture |
|  | W10 | Fall on and from stairs and steps |
|  | W18 | Other fall on same level |
|  | W19 | Unspecified fall |
|  | R29.6 | Tendency to fall, not elsewhere classified |
|  | E880 | fall on stairs or steps* |
|  | E880.0 | fall on escalator |
|  | E880.1 | fall on sidewalk curb |
|  | E880.9 | fall on stair/step nec |
|  | E884.2 | fall from chair |
|  | E884.3 | fall from wheelchair |
|  | E884.4 | fall from bed |
|  | E884.5 | fall from furniture nec |
|  | E884.6 | fall from commode |
|  | E885 | fall on level-tripping* |
|  | E885.9 | fall from slipping nec |
|  | E888 | fall nec & nos* |
|  | E888.8 | fall nec |
|  | E888.9 | fall nos |
| **Hypotension** | 458 | hypotension* |
|  | 458.0 | orthostatic hypotension |
|  | 458.1 | chronic hypotension |
|  | 458.8 | hypotension nec |
|  | 458.9 | hypotension nos |
|  | I95 | Hypotension |
|  | I95.0 | Idiopathic hypotension |
|  | I95.1 | Orthostatic hypotension |
|  | I95.2 | Hypotension due to drugs |
|  | I95.8 | Other hypotension |
|  | I95.9 | Hypotension, unspecified |
| **Syncope** | R55 | Syncope and collapse |
|  | 780.2 | Syncope and collapse |
| **Fracture** | T14.2 | Fracture of unspecified body region |
|  | T12 | Fracture of lower limb, level unspecified |
|  | T10 | Fracture of upper limb, level unspecified |
|  | T08 | Fracture of spine, level unspecified |
|  | T02.9 | Multiple fractures, unspecified |
|  | T02.8 | Fractures involving other combinations of body regions |
|  | T02.7 | Fractures involving thorax with lower back and pelvis with limb(s) |
|  | T02.6 | Fractures involving multiple regions of upper limb(s) with lower limb(s) |
|  | T02.5 | Fractures involving multiple regions of both lower limbs |
|  | T02.4 | Fractures involving multiple regions of both upper limbs |
|  | T02.3 | Fractures involving multiple regions of one lower limb |
|  | T02.2 | Fractures involving multiple regions of one upper limb |
|  | T02.1 | Fractures involving thorax with lower back and pelvis |
|  | T02.0 | Fractures involving head with neck |
|  | T02 | Fractures involving multiple body regions |
|  | S92.9 | Fracture of foot, unspecified |
|  | S92.7 | Multiple fractures of foot |
|  | S92.5 | Fracture of other toe |
|  | S92.4 | Fracture of great toe |
|  | S92.3 | Fracture of metatarsal bone |
|  | S92.2 | Fracture of other tarsal bone(s) |
|  | S92.1 | Fracture of talus |
|  | S92.0 | Fracture of calcaneus |
|  | S92 | Fracture of foot, except ankle |
|  | S82.9 | Fracture of lower leg, part unspecified |
|  | S82.8 | Fractures of other parts of lower leg |
|  | S82.7 | Multiple fractures of lower leg |
|  | S82.6 | Fracture of lateral malleolus |
|  | S82.5 | Fracture of medial malleolus |
|  | S82.4 | Fracture of fibula alone |
|  | S82.3 | Fracture of lower end of tibia |
|  | S82.2 | Fracture of shaft of tibia |
|  | S82.1 | Fracture of upper end of tibia |
|  | S82.0 | Fracture of patella |
|  | S82 | Fracture of lower leg, including ankle |
|  | S72.9 | Fracture of femur, part unspecified |
|  | S72.8 | Fractures of other parts of femur |
|  | S72.7 | Multiple fractures of femur |
|  | S72.4 | Fracture of lower end of femur |
|  | S72.3 | Fracture of shaft of femur |
|  | S72.2 | Subtrochanteric fracture |
|  | S72.1 | Pertrochanteric fracture |
|  | S72.0 | Fracture of neck of femur |
|  | S72 | Fracture of femur |
|  | S62.8 | Fracture of other and unspecified parts of wrist and hand |
|  | S62.7 | Multiple fractures of fingers |
|  | S62.6 | Fracture of other finger |
|  | S62.5 | Fracture of thumb |
|  | S62.4 | Multiple fractures of metacarpal bones |
|  | S62.3 | Fracture of other metacarpal bone |
|  | S62.2 | Fracture of first metacarpal bone |
|  | S62.1 | Fracture of other carpal bone(s) |
|  | S62.0 | Fracture of navicular [scaphoid] bone of hand |
|  | S62 | Fracture at wrist and hand level |
|  | S52.9 | Fracture of forearm, part unspecified |
|  | S52.8 | Fracture of other parts of forearm |
|  | S52.7 | Multiple fractures of forearm |
|  | S52.6 | Fracture of lower end of both ulna and radius |
|  | S52.5 | Fracture of lower end of radius |
|  | S52.4 | Fracture of shafts of both ulna and radius |
|  | S52.3 | Fracture of shaft of radius |
|  | S52.2 | Fracture of shaft of ulna |
|  | S52.1 | Fracture of upper end of radius |
|  | S52.0 | Fracture of upper end of ulna |
|  | S52 | Fracture of forearm |
|  | S42.9 | Fracture of shoulder girdle, part unspecified |
|  | S42.8 | Fracture of other parts of shoulder and upper arm |
|  | S42.7 | Multiple fractures of clavicle, scapula and humerus |
|  | S42.4 | Fracture of lower end of humerus |
|  | S42.3 | Fracture of shaft of humerus |
|  | S42.2 | Fracture of upper end of humerus |
|  | S42.1 | Fracture of scapula |
|  | S42.0 | Fracture of clavicle |
|  | S42 | Fracture of shoulder and upper arm |
|  | S32.8 | Fracture of other and unspecified parts of lumbar spine and pelvis |
|  | S32.7 | Multiple fractures of lumbar spine and pelvis |
|  | S32.5 | Fracture of pubis |
|  | S32.4 | Fracture of acetabulum |
|  | S32.3 | Fracture of ilium |
|  | S32.2 | Fracture of coccyx |
|  | S32.1 | Fracture of sacrum |
|  | S32.0 | Fracture of lumbar vertebra |
|  | S32 | Fracture of lumbar spine and pelvis |
|  | S22.9 | Fracture of bony thorax, part unspecified |
|  | S22.8 | Fracture of other parts of bony thorax |
|  | S22.4 | Multiple fractures of ribs |
|  | S22.3 | Fracture of rib |
|  | S22.2 | Fracture of sternum |
|  | S22.1 | Multiple fractures of thoracic spine |
|  | S22.0 | Fracture of thoracic vertebra |
|  | S22 | Fracture of rib(s), sternum and thoracic spine |
|  | S12.9 | Fracture of neck, part unspecified |
|  | S12.8 | Fracture of other parts of neck |
|  | S12.7 | Multiple fractures of cervical spine |
|  | S12.2 | Fracture of other specified cervical vertebra |
|  | S12.1 | Fracture of second cervical vertebra |
|  | S12.0 | Fracture of first cervical vertebra |
|  | S12 | Fracture of neck |
|  | S02.9 | Fracture of skull and facial bones, part unspecified |
|  | S02.8 | Fractures of other skull and facial bones |
|  | S02.7 | Multiple fractures involving skull and facial bones |
|  | S02.6 | Fracture of mandible |
|  | S02.5 | Fracture of tooth |
|  | S02.4 | Fracture of malar and maxillary bones |
|  | S02.3 | Fracture of orbital floor |
|  | S02.2 | Fracture of nasal bones |
|  | S02.1 | Fracture of base of skull |
|  | S02.0 | Fracture of vault of skull |
|  | S02 | Fracture of skull and facial bones |
|  | E887 | fracture, cause nos |
|  | 829.1 | fracture nos-open |
|  | 829.0 | fracture nos-closed |
|  | 829 | fracture nos* |
|  | 828.1 | fx legs w arm/rib-open |
|  | 828.0 | fx legs w arm/rib-closed |
|  | 828 | fx legs w arm/rib* |
|  | 827.1 | fx lower limb nec-open |
|  | 827.0 | fx lower limb nec-closed |
|  | 827 | lower limb fracture nec* |
|  | 826.1 | fx phalanx, foot-open |
|  | 826.0 | fx phalanx, foot-closed |
|  | 826 | fracture phalanges, foot* |
|  | 825.3 | fx tars/metatars nec-opn* |
|  | 825.2 | fx tarsl/metatars nec-cl* |
|  | 825.1 | fracture calcaneus-open |
|  | 825.0 | fracture calcaneus-close |
|  | 825 | fx of tarsal/metatarsal* |
|  | 824.9 | fx ankle nos-open |
|  | 824.8 | fx ankle nos-closed |
|  | 824.7 | fx trimalleolar-open |
|  | 824.6 | fx trimalleolar-closed |
|  | 824.5 | fx bimalleolar-open |
|  | 824.4 | fx bimalleolar-closed |
|  | 824.3 | fx lateral malleolus-opn |
|  | 824.2 | fx lateral malleolus-cl |
|  | 824.1 | fx medial malleolus-open |
|  | 824.0 | fx medial malleolus-clos |
|  | 824 | ankle fracture* |
|  | 823.9 | fx tibia/fibula nos-open* |
|  | 823.8 | fx tibia/fibula nos-clos* |
|  | 823.4 | * |
|  | 823.3 | fx tibia/fibula shaft-op* |
|  | 823.2 | fx shaft tib/fib-closed* |
|  | 823.1 | fx upper tibia/fibula-op* |
|  | 823.0 | fx upper tibia/fibula-cl* |
|  | 823 | tibia & fibula fracture* |
|  | 822.1 | fracture patella-open |
|  | 822.0 | fracture patella-closed |
|  | 822 | patella fracture* |
|  | 821.3 | fx lower end femur-open* |
|  | 821.2 | fx lower end femur-close* |
|  | 821.1 | fx femur shaft/nos-open* |
|  | 821.0 | fx femur shaft/nos-close* |
|  | 821 | other femoral fracture* |
|  | 820.9 | fx neck of femur nos-opn |
|  | 820.8 | fx neck of femur nos-cl |
|  | 820.3 | pertrochanteric fx-open* |
|  | 820.2 | pertrochanteric fx-clos* |
|  | 820.1 | transcerv fx femur-open* |
|  | 820.0 | transcerv fx femur-close* |
|  | 820 | fracture neck of femur* |
|  | 819.1 | fx arms w rib/stern-open |
|  | 819.0 | fx arms w rib/sternum-cl |
|  | 819 | fx arms w rib/sternum* |
|  | 818.1 | fx arm mult/nos-open |
|  | 818.0 | fx arm mult/nos-closed |
|  | 818 | fracture arm mult/nos* |
|  | 817.1 | multiple fx hand-open |
|  | 817.0 | multiple fx hand-closed |
|  | 817 | multiple hand fractures* |
|  | 816.1 | fx phalanges, hand-open* |
|  | 816.0 | fx phalanges, hand-close* |
|  | 816 | fracture phalanges, hand* |
|  | 815.1 | fracture metacarpal-open* |
|  | 815.0 | fracture metacarpal-clos* |
|  | 815 | metacarpal fracture* |
|  | 814.1 | fracture carpal bone-opn* |
|  | 814.0 | fracture carpal bone-cl* |
|  | 814 | carpal fracture* |
|  | 813.9 | fx radius/ulna nos-open* |
|  | 813.8 | fx radius/ulna nos-close* |
|  | 813.5 | fx low radius w/ulna-opn* |
|  | 813.4 | fx lower radius/ulna-cl* |
|  | 813.3 | fx radius/ulna shaft-opn* |
|  | 813.2 | fx radius/ulna shaft-cl* |
|  | 813.1 | fx upper radius/ulna-opn* |
|  | 813.0 | fx upper radius/ulna-cl* |
|  | 813 | radius & ulna fracture* |
|  | 812.5 | fx lower humerus-open* |
|  | 812.4 | fx lower humerus-closed* |
|  | 812.3 | fx humerus shaft/nos-opn* |
|  | 812.2 | fx humerus shaft/nos-cl* |
|  | 812.1 | fx upper humerus-open* |
|  | 812.0 | fx upper humerus-closed* |
|  | 812 | humerus fracture* |
|  | 811.1 | fracture of scapula-open* |
|  | 811.0 | fracture scapula-closed* |
|  | 811 | scapula fracture* |
|  | 810.1 | fracture clavicle-open* |
|  | 810.0 | fracture clavicle-closed* |
|  | 810 | clavicle fracture* |
|  | 809.1 | fracture trunk bone-open |
|  | 809.0 | fracture trunk bone-clos |
|  | 809 | fracture of trunk bones* |
|  | 808.9 | pelvic fracture nos-open |
|  | 808.8 | pelvic fracture nos-clos |
|  | 808.5 | oth pelvic fracture-open* |
|  | 808.4 | oth pelvic fracture-clos* |
|  | 808.3 | fracture of pubis-open |
|  | 808.2 | fracture of pubis-closed |
|  | 808.1 | fracture acetabulum-open |
|  | 808.0 | fracture acetabulum-clos |
|  | 808 | pelvic fracture* |
|  | 807.6 | fx larynx/trachea-open |
|  | 807.5 | fx larynx/trachea-closed |
|  | 807.4 | flail chest |
|  | 807.3 | fracture of sternum-open |
|  | 807.2 | fracture of sternum-clos |
|  | 807.1 | fracture of rib-open* |
|  | 807.0 | fracture of rib-closed* |
|  | 807 | fx rib/stern/laryn/trach* |
|  | 806.9 | vert fx nos-op w crd inj |
|  | 806.8 | vert fx nos-cl w crd inj |
|  | 806.7 | fx sacrum-opn w cord inj* |
|  | 806.6 | fx sacrum-cl w cord inj* |
|  | 806.5 | opn lumbar fx w cord inj |
|  | 806.4 | cl lumbar fx w cord inj |
|  | 806.3 | opn dorsal fx w cord inj* |
|  | 806.2 | cl dorsal fx w cord inj* |
|  | 806.1 | open cerv fx w cord inj* |
|  | 806.0 | clos cerv fx w cord inj* |
|  | 806 | vertebral fx w cord inj* |
|  | 805.9 | vertebral fx nos-open |
|  | 805.8 | vertebral fx nos-closed |
|  | 805.7 | fx sacrum/coccyx-open |
|  | 805.6 | fx sacrum/coccyx-closed |
|  | 805.5 | fx lumbar vertebra-open |
|  | 805.4 | fx lumbar vertebra-close |
|  | 805.3 | fx dorsal vertebra-open |
|  | 805.2 | fx dorsal vertebra-close |
|  | 805.1 | fx cervical vertebra-opn* |
|  | 805.0 | fx cervical vertebra-cl* |
|  | 805 | vertebrl fx w/o cord inj* |
|  | 804.9 | op skl/oth fx/br inj nec* |
|  | 804.8 | opn skl w oth fx/hem nec* |
|  | 804.7 | opn skl/oth fx/menin hem* |
|  | 804.6 | opn skl/oth fx/cereb lac* |
|  | 804.5 | opn skull fx/oth bone fx* |
|  | 804.4 | cl skl/oth fx/br inj nec* |
|  | 804.3 | cl skul w oth fx/hem nec* |
|  | 804.2 | cl skl/oth fx/mening hem* |
|  | 804.1 | cl sk w oth fx/cereb lac* |
|  | 804.0 | cl skul fx w oth bone fx* |
|  | 804 | mult fx skull w oth bone* |
|  | 803.9 | op skl fx nec/br inj nec* |
|  | 803.8 | opn skull fx nec/hem nec* |
|  | 803.7 | opn skl fx nec/menin hem* |
|  | 803.6 | opn skl fx nec/cereb lac* |
|  | 803.5 | open skull fracture nec* |
|  | 803.4 | cl skl fx nec/br inj nec* |
|  | 803.3 | cl skull fx nec/hem nec* |
|  | 803.2 | cl skl fx nec/mening hem* |
|  | 803.1 | cl skl fx nec/cerebr lac* |
|  | 803.0 | close skull fracture nec* |
|  | 803 | other skull fracture* |
|  | 802.9 | fx facial bone nec-open |
|  | 802.8 | fx facial bone nec-close |
|  | 802.7 | fx orbital floor-open |
|  | 802.6 | fx orbital floor-closed |
|  | 802.5 | fx malar/maxillary-open |
|  | 802.4 | fx malar/maxillary-close |
|  | 802.3 | mandible fracture-open* |
|  | 802.2 | mandible fracture-closed* |
|  | 802.1 | nasal bone fx-open |
|  | 802.0 | nasal bone fx-closed |
|  | 802 | fracture of face bones* |
|  | 801.9 | op sk base fx/br inj nec* |
|  | 801.8 | opn skul base fx/hem nec* |
|  | 801.7 | op skl base fx/menin hem* |
|  | 801.6 | op skl base fx/cereb lac* |
|  | 801.5 | open skull base fracture* |
|  | 801.4 | cl sk base fx/br inj nec* |
|  | 801.3 | cl skull base fx/hem nec* |
|  | 801.2 | cl skl base fx/menin hem* |
|  | 801.1 | cl skl base fx/cereb lac* |
|  | 801.0 | clos skull base fracture* |
|  | 801 | skull base fracture* |
|  | 800.9 | op skl vlt fx/br inj nec* |
|  | 800.8 | opn skull vlt fx/hem nec* |
|  | 800.7 | opn skl vlt fx/menin hem* |
|  | 800.6 | opn skl vlt fx/cereb lac* |
|  | 800.5 | opn skull vault fracture* |
|  | 800.4 | cl skl vlt fx/br inj nec* |
|  | 800.3 | cl skull vlt fx/hem nec* |
|  | 800.2 | cl skl vlt fx/mening hem* |
|  | 800.1 | cl skl vlt fx/cerebr lac* |
|  | 800.0 | closed skull vault fx* |
|  | 800 | skull vault fracture* |
| **Acute kidney injury** | S37.0 | Injury of kidney |
|  | N19 | Unspecified kidney failure |
|  | N17 | Acute renal failure |
|  | N17.0 | Acute renal failure with tubular necrosis |
|  | N17.1 | Acute renal failure with acute cortical necrosis |
|  | N17.2 | Acute renal failure with medullary necrosis |
|  | N17.8 | Other acute renal failure |
|  | N17.9 | Acute renal failure, unspecified |
|  | 866 | kidney injury* |
|  | 866.0 | kidney injury-closed* |
|  | 866.00 | kidney injury nos-closed |
|  | 586 | renal failure nos |
|  | 584 | acute renal failure* |
|  | 584.5 | ac kidny fail, tubr necr |
|  | 584.6 | ac kidny fail, cort necr |
|  | 584.7 | ac kidny fail, medu necr |
|  | 584.8 | acute kidney failure nec |
|  | 584.9 | acute kidney failure nos |
| **Electrolyte abnormalities** | 276 | fluid/electrolyte dis* |
|  | 276.0 | hyperosmolality |
|  | 276.1 | hyposmolality |
|  | 276.2 | acidosis |
|  | 276.3 | alkalosis |
|  | 276.4 | mixed acid-base bal dis |
|  | 276.5 | hypovolemia# |
|  | 276.6 | fluid overload# |
|  | 276.7 | hyperpotassemia |
|  | 276.8 | hypopotassemia |
|  | 276.9 | electrolyt/fluid dis nec |
|  | E87 | Other disorders of fluid, electrolyte and acid-base balance |
|  | E87.0 | Hyperosmolality and hypernatraemia |
|  | E87.1 | Hypo-osmolality and hyponatraemia |
|  | E87.2 | Acidosis |
|  | E87.3 | Alkalosis |
|  | E87.4 | Mixed disorder of acid-base balance |
|  | E87.5 | Hyperkalaemia |
|  | E87.6 | Hypokalaemia |
|  | E87.7 | Fluid overload |
|  | E87.8 | Other disorders of electrolyte and fluid balance, not elsewhere classified |
| **Gout** (based on primary care records only) | **Read code** | **Description** |
|  | 1443.00 | H/O: gout |
|  | 669..00 | Gout monitoring |
|  | 6693.00 | Joints gout affected |
|  | 6695.00 | Date gout treatment started |
|  | 6696.00 | Date of last gout attack |
|  | 6697.00 | Gout associated problems |
|  | 6698.00 | Gout drug side effects |
|  | 6699.00 | Gout treatment changed |
|  | 669A.00 | Date gout treatment stopped |
|  | 669Z.00 | Gout monitoring NOS |
|  | C34..00 | Gout |
|  | C340.00 | Gouty arthropathy |
|  | C341.00 | Gouty nephropathy |
|  | C341z00 | Gouty nephropathy NOS |
|  | C342.00 | Idiopathic gout |
|  | C343.00 | Lead-induced gout |
|  | C344.00 | Drug-induced gout |
|  | C345.00 | Gout due to impairment of renal function |
|  | C346.00 | Acute exacerbation of gout |
|  | C34y.00 | Other specified gouty manifestation |
|  | C34y000 | Gouty tophi of ear |
|  | C34y100 | Gouty tophi of heart |
|  | C34y200 | Gouty tophi of other sites |
|  | C34y300 | Gouty iritis |
|  | C34y400 | Gouty neuritis |
|  | C34y500 | Gouty tophi of hand |
|  | C34yz00 | Other specified gouty manifestation NOS |
|  | C34z.00 | Gout NOS |
|  | G557300 | Gouty tophi of heart |
|  | N02..14 | Pseudogout |
|  | N023.00 | Gouty arthritis |
|  | N023100 | Gouty arthritis of the shoulder region |
|  | N023200 | Gouty arthritis of the upper arm |
|  | N023300 | Gouty arthritis of the forearm |
|  | N023400 | Gouty arthritis of the hand |
|  | N023600 | Gouty arthritis of the lower leg |
|  | N023700 | Gouty arthritis of the ankle and foot |
|  | N023800 | Gouty arthritis of toe |
|  | N023x00 | Gouty arthritis of multiple sites |
|  | N023y00 | Gouty arthritis of other specified site |
|  | N023z00 | Gouty arthritis NOS |
|  | Nyu1700 | [X]Other secondary gout |
