# Supplementary material for: The association between antihypertensive treatment and serious adverse events by age and frailty: A cohort study
Source: PLoS Med. 2023 Apr 19;20(4):e1004223. doi: 10.1371/journal.pmed.1004223 (PMC10155987; doi:10.1371/journal.pmed.1004223)
Supplement: S2 Table — (DOCX) [file pmed.1004223.s007.docx]

**S2 Table.** Drug types and corresponding British National Formulary (BNF) header included in the analysis

| **Drug group** | **Drug** |
| --- | --- |
| **Antihypertensive** | Acebutolol hydrochloride |
|  | Aliskiren hemifumarate |
|  | Ambrisentan |
|  | Amlodipine |
|  | Atenolol |
|  | Azilsartan medoxomil |
|  | Bendroflumethiazide |
|  | Bisoprolol Fumarate |
|  | Bosentan monohydrate |
|  | Candesartan cilexetil |
|  | Captopril |
|  | Carteolol Hydrochloride |
|  | Carvedilol |
|  | Celiprolol hydrochloride |
|  | Chlortalidone |
|  | Clonidine |
|  | Diazoxide |
|  | Diltiazem Hydrochloride |
|  | Doxazosin mesilate |
|  | Enalapril Maleate |
|  | Eprosartan mesilate |
|  | Esmolol Hydrochloride |
|  | Felodipine |
|  | Fosinopril sodium |
|  | Hydralazine Hydrochloride |
|  | Hydrochlorothiazide |
|  | Iloprost trometamol |
|  | Imidapril hydrochloride |
|  | Indapamide |
|  | Indoramin Hydrochloride |
|  | Irbesartan |
|  | Isradipine |
|  | Labetalol hydrochloride |
|  | Lacidipine |
|  | Lercanidipine hydrochloride |
|  | Lidoflazine |
|  | Lisinopril |
|  | Losartan potassium |
|  | Macitentan |
|  | Meprobamate/Bendroflumethiazide |
|  | Methyldopa Anhydrous |
|  | Methyldopate Hydrochloride |
|  | Metolazone |
|  | Metoprolol |
|  | Metoprolol Tartrate |
|  | Mibefradil |
|  | Minoxidil |
|  | Moexipril hydrochloride |
|  | Moxonidine |
|  | Nadolol |
|  | Nebivolol hydrochloride |
|  | Nicardipine hydrochloride |
|  | Nifedipine |
|  | Nimodipine |
|  | Nisoldipine |
|  | Olmesartan medoxomil |
|  | Oxprenolol Hydrochloride |
|  | Oxprenolol Hydrochloride/Cyclopenthiazide |
|  | Perhexiline Maleate |
|  | Perindopril Erbumine |
|  | Perindopril arginine |
|  | Perindopril tosilate |
|  | Phentolamine mesilate |
|  | Pindolol |
|  | Pindolol/Clopamide |
|  | Potassium Chloride/Chlortalidone |
|  | Practolol |
|  | Prazosin Hydrochloride |
|  | Prenylamine Lactate |
|  | Propranolol Hydrochloride |
|  | Quinapril Hydrochloride |
|  | Quinapril hydrochloride |
|  | Ramipril |
|  | Reserpine/Bendroflumethiazide |
|  | Reserpine/Hydrochlorothiazide |
|  | Riociguat |
|  | Sacubitril/Valsartan |
|  | Sildenafil Citrate |
|  | Sodium nitroprusside dihydrate |
|  | Sotalol Hydrochloride/Hydrochlorothiazide |
|  | Tadalafil |
|  | Telmisartan |
|  | Terazosin hydrochloride |
|  | Terazosine |
|  | Timolol maleate |
|  | Timolol maleate/Amiloride hydrochlori.. |
|  | Trandolapril |
|  | Triamterene/Chlortalidone |
|  | Valsartan |
|  | Verapamil hydrochloride |
|  | Xipamide |
| **Anticholinergics** | Amantadine hydrochloride |
|  | Amitriptyline hydrochloride/Perphenazine |
|  | Aspirin/Methocarbamol |
|  | Atropine/Isoprenaline Hydrochloride |
|  | Atropine/Papaverine Hydrochloride/Adrenaline |
|  | Atropine/Papaverine Hydrochloride/Adrenaline Acid Tartrate |
|  | Atropine/Papaverine Hydrochloride/Adrenaline Hydrochloride/Benzocaine |
|  | Atropine sulfate |
|  | Benzatropine mesilate |
|  | Brompheniramine maleate |
|  | Chlorpromazine embonate |
|  | Chlorpromazine Hydrochloride |
|  | Cinnarizine/Dimenhydrinate |
|  | Clozapine |
|  | Darifenacin hydrobromide |
|  | Dimenhydrinate |
|  | Diphenoxylate Hydrochloride/Atropine Sulphate |
|  | Fesoterodine fumarate |
|  | Flavoxate Hydrochloride |
|  | Guaifenesin/Pseudoephedrine hydrochloride/Brompheniramine maleate |
|  | Homatropine hydrobromide |
|  | Isopropamide Iodide/Trifluoperazine Hydrochloride |
|  | Methocarbamol |
|  | Nefopam hydrochloride |
|  | Olanzapine |
|  | Orphenadrine hydrochloride |
|  | Oxcarbazepine |
|  | Oxybutynin |
|  | Paracetamol/Orphenadrine Hydrochloride |
|  | Paroxetine hydrochloride |
|  | Perphenazine |
|  | Propantheline bromide |
|  | Propiverine hydrochloride |
|  | Pseudoephedrine Hydrochloride/Brompheniramine Maleate |
|  | Quetiapine fumarate |
|  | Solifenacin succinate |
|  | Thioridazine |
|  | Tolterodine tartrate |
|  | Trifluoperazine hydrochloride |
|  | Trospium chloride |
| **Antidepressants** | Amitriptyline hydrochloride |
|  | Buspirone hydrochloride |
|  | Clomipramine hydrochloride |
|  | Dosulepin hydrochloride |
|  | Doxepin hydrochloride |
|  | Duloxetine hydrochloride |
|  | Escitalopram oxalate |
|  | Imipramine hydrochloride |
|  | Isocarboxazid |
|  | Lofepramine hydrochloride |
|  | Mianserin hydrochloride |
|  | Mirtazapine |
|  | Moclobemide |
|  | Nortriptyline hydrochloride |
|  | Paroxetine hydrochloride |
|  | Phenelzine sulfate |
|  | Sertraline |
|  | Sertraline hydrochloride |
|  | Tranylcypromine sulfate |
|  | Trazodone hydrochloride |
|  | Trifluoperazine Hydrochloride/Tranylcypromine Sulphate |
|  | Trimipramine maleate |
|  | Venlafaxine hydrochloride |
|  | Vortioxetine hydrobromide |
|  | Alprazolam |
| **Hypnotics, anxiolytics** | Buspirone hydrochloride |
|  | Chlordiazepoxide Hydrochloride |
|  | Clobazam |
|  | Clomethiazole |
|  | Diazepam |
|  | Flunitrazepam |
|  | Flurazepam hydrochloride |
|  | Hydroxyzine hydrochloride |
|  | Loprazolam mesilate |
|  | Lorazepam |
|  | Lormetazepam |
|  | Meprobamate |
|  | Nitrazepam |
|  | Oxazepam |
|  | Promethazine hydrochloride |
|  | Temazepam |
|  | Thiopental sodium |
|  | Zolpidem tartrate |
|  | Zopiclone |
|  | Alfentanil Hydrochloride |
| **Opioids** | Buprenorphine |
|  | Codeine phosphate |
|  | Cyclizine Tartrate/Morphine Tartrate |
|  | Diamorphine hydrochloride |
|  | Dihydrocodeine Tartrate |
|  | Ethylmorphine hydrochloride |
|  | Fentanyl |
|  | Meptazinol hydrochloride |
|  | Methadone hydrochloride |
|  | Morphine Anhydrous |
|  | Morphine hydrochloride |
|  | Morphine Sulphate |
|  | Naloxone hydrochloride |
|  | Oxycodone hydrochloride |
|  | Pentazocine hydrochloride |
|  | Pentazocine lactate |
|  | Pethidine hydrochloride |
|  | Remifentanyl hydrochloride |
|  | Tapentadol hydrochloride |
|  | Tramadol hydrochloride |
| **Statins** | Atorvastatin calcium trihydrate |
|  | Cerivastatin sodium |
|  | Fluvastatin sodium |
|  | Pravastatin sodium |
|  | Rosuvastatin calcium |
|  | Simvastatin |
|  | Abciximab |
| **Anti-thrombotics** | Acenocoumarol |
|  | Apixaban |
|  | Aspirin |
|  | Bivalirudin |
|  | Certoparin Sodium |
|  | Clopidogrel |
|  | Dabigatran etexilate mesilate |
|  | Dalteparin sodium |
|  | Danaparoid sodium |
|  | Dipyridamole |
|  | Edoxaban tosilate |
|  | Enoxaparin sodium |
|  | Epoprostenol sodium |
|  | Fondaparinux sodium |
|  | Heparin |
|  | Lepirudin |
|  | Pentosan polysulfate sodium |
|  | Phenindione |
|  | Prasugrel hydrochloride |
|  | Rivaroxaban |
|  | Sodium citrate/Citric acid monohydrate/Glucose monohydrate |
|  | Ticagrelor |
|  | Ticlopidine hydrochloride |
|  | Tinzaparin sodium |
|  | Tirofiban |
|  | Warfarin sodium |
