# Supplementary material for: The association between antihypertensive treatment and serious adverse events by age and frailty: A cohort study
Source: PLoS Med. 2023 Apr 19;20(4):e1004223. doi: 10.1371/journal.pmed.1004223 (PMC10155987; doi:10.1371/journal.pmed.1004223)
Supplement: S3 Table — *Proportions based on the number of patients with data available (i.e., excluding those with missing values) †IMD, indices of multiple deprivation; IMD score of 5 indicates patients in the highest quintile of deprivation (most deprived). (DOCX) [file pmed.1004223.s008.docx]

**S3 Table.** Baseline characteristics within each dataset (CPRD Gold vs CPRD Aurum)

| Characteristic | CPRD Gold population | | CPRD Aurum population | |
| --- | --- | --- | --- | --- |
|  | **Mean/number** | **SD/%** | **Mean/number** | **SD/%** |
| Total population | 1,219,732 |  | 2,614,324 |  |
| Age (years) (SD) | 57.2 | 12.4 | 56.3 | 12.3 |
| Sex (% female) | 606,001 | 49.7% | 1,305,801 | 49.9% |
| White ethnicity (%)* | 430,285 | 94.7% | 1,317,091 | 63.1% |
| Black ethnicity (%)* | 5,970 | 1.3% | 78,045 | 3.7% |
| South Asian ethnicity (%)* | 8,521 | 1.9% | 64,286 | 3.1% |
| Other ethnicity (%)* | 9,641 | 2.1% | 627,889 | 30.1% |
| High deprivation (IMD score of 5) (%)*† | 166,814 | 13.7% | 396,860 | 16.8% |
| Current smoking status (%)* | 264,376 | 22.8% | 604,431 | 24.6% |
| Alcohol consumption (heavy drinker) (%)* | 16,574 | 2.0% | 51,786 | 2.6% |
| Body mass index (kg/m2) (SD) | 27.1 | 5.2 | 27.3 | 5.3 |
| Systolic blood pressure (mmHg) (SD) | 142.6 | 11.6 | 142.6 | 11.7 |
| Diastolic blood pressure (mmHg) (SD) | 83.9 | 9.4 | 83.8 | 9.5 |
| QRisk2 risk score (SD) | 12.7% | 12.1% | 12.1% | 12.3% |
| eFrailty index score (SD) | 0.04 | 0.05 | 0.05 | 0.05 |
| Co-morbidities |  |  |  |  |
| Stroke (%) | 17,957 | 1.5% | 43,245 | 1.7% |
| Transient ischemic attack (%) | 8,355 | 0.7% | 20,358 | 0.8% |
| Myocardial infarction (%) | 13,596 | 1.1% | 30,857 | 1.2% |
| Heart failure (%) | 7,310 | 0.6% | 18,590 | 0.7% |
| Peripheral vascular disease (%) | 5,617 | 0.5% | 16,784 | 0.6% |
| Coronary artery bypass graft (%) | 2,630 | 0.2% | 6,944 | 0.3% |
| Angina (%) | 20,072 | 1.6% | 38,100 | 1.5% |
| Atrial fibrillation (%) | 18,607 | 1.5% | 40,162 | 1.5% |
| Diabetes (%) | 65,447 | 5.4% | 156,208 | 6.0% |
| Chronic kidney disease (%) | 13,534 | 1.1% | 35,994 | 1.4% |
| Cancer (%) | 44,631 | 3.7% | 96,261 | 3.7% |
| Treatment prescriptions |  |  |  |  |
| ACE inhibitors (%) | 59,453 | 4.9% | 127,756 | 4.9% |
| Angiotensin II receptor blockers (%) | 14,724 | 1.2% | 33,505 | 1.3% |
| Calcium channel blockers (%) | 43,735 | 3.6% | 97,719 | 3.7% |
| Thiazides and thiazide-like diuretics (%) | 47,066 | 3.9% | 101,586 | 3.9% |
| Beta-blockers (%) | 52,300 | 4.3% | 109,911 | 4.2% |
| Alpha-blockers (%) | 6,744 | 0.6% | 13,330 | 0.5% |
| Other antihypertensives (%) | 2,884 | 0.2% | 5,259 | 0.2% |
| Statins (%) | 114,162 | 9.4% | 252,569 | 9.7% |
| Anti-thrombotics (%) | 120,408 | 9.9% | 255,388 | 9.8% |
| Anticholinergics (%) | 115,290 | 9.5% | 219,811 | 8.4% |
| Antidepressants (%) | 223,177 | 18.3% | 469,792 | 18.0% |
| Hypnotics/anxiolytics (%) | 218,821 | 17.9% | 443,358 | 17.0% |
| Opioids (%) | 330,504 | 27.1% | 741,029 | 28.3% |

*Proportions based on the number of patients with data available (i.e. excluding those with missing values)

†IMD = indices of multiple deprivation; IMD score of 5 indicates patients in the highest quintile of deprivation (most deprived)
