# Supplementary material for: The association between antihypertensive treatment and serious adverse events by age and frailty: A cohort study
Source: PLoS Med. 2023 Apr 19;20(4):e1004223. doi: 10.1371/journal.pmed.1004223 (PMC10155987; doi:10.1371/journal.pmed.1004223)
Supplement: S4 Table — ACE, angiotensin-converting enzyme; IQR, interquartile range; SD, standard deviation. Data are based on the primary analysis examining and censoring fall events. (DOCX) [file pmed.1004223.s009.docx]

**S4 Table.** Antihypertensive drug exposure during follow-up in the study

| Medication prescription after the index date (during follow-up) | No antihypertensive prescription during 12 month exposure period (non-exposed; n=3,349,869) | | Antihypertensive prescription during 12 month exposure period (exposed; n=484,187) | |
| --- | --- | --- | --- | --- |
|  | **Number/ median** | **%/IQR/SD** | **Number/ median** | **%/IQR/SD** |
| Total prescribed antihypertensives (%) | 936,455 | 28% | 431,599 | 89% |
| ACE inhibitors (%) | 281,867 | 8% | 160,572 | 33% |
| Angiotensin II receptor blockers (%) | 17,753 | 1% | 42,248 | 9% |
| Calcium channel blockers (%) | 174,912 | 5% | 70,401 | 15% |
| Thiazides and thiazide-like diuretics (%) | 152,034 | 5% | 55,414 | 11% |
| Beta-blockers (%) | 206,259 | 6% | 91,655 | 19% |
| Alpha-blockers (%) | 10,933 | <1% | 7,917 | 2% |
| Other antihypertensives (%) | 92,697 | 3% | 3,392 | 1% |
| Average number of years exposed to treatment [median, IQR] | 0.0 | 0 to 0.8 | 6.0 | 2.0 to 10.0 |
| Average number of years exposed to treatment [mean, SD] | 1.3 | 2.6 | 5.7 | 3.8 |

IQR=inter-quartile range; SD=standard deviation; ACE=angiotensin-converting-enzyme

Data are based on the primary analysis examining and censoring fall events.
