# Supplementary material for: The association between antihypertensive treatment and serious adverse events by age and frailty: A cohort study
Source: PLoS Med. 2023 Apr 19;20(4):e1004223. doi: 10.1371/journal.pmed.1004223 (PMC10155987; doi:10.1371/journal.pmed.1004223)
Supplement: S5 Table — CI, confidence interval; DBP, diastolic blood pressure; HDL, high-density lipoprotein; IMD, indices of multiple deprivation; SBP, systolic blood pressure. (DOCX) [file pmed.1004223.s010.docx]

**S5 Table.** Propensity score model

| **Variable** | | **Odds ratio** | **95% CI** | | |
| --- | --- | --- | --- | --- | --- |
| Patient characteristic | Age (<65 years vs ≥65 years) | 0.62 | 0.62 | - | 0.63 |
|  | Sex (ref male) | 1.49 | 1.47 | - | 1.50 |
| Ethnicity  (ref white) | Black | 2.14 | 2.10 | - | 2.19 |
|  | South Asian | 1.17 | 1.14 | - | 1.20 |
|  | Other | 1.16 | 1.14 | - | 1.17 |
| Smoking status  (ref non-smoker) | Ex-smoker | 1.02 | 1.01 | - | 1.03 |
|  | Current smoker | 0.79 | 0.78 | - | 0.80 |
| Alcohol consumption  (ref non-drinker) | Trivial drinker (<1 unit/day) | 0.95 | 0.94 | - | 0.96 |
|  | Light drinker (1-2 units/day) | 0.97 | 0.96 | - | 0.98 |
|  | Moderate drinker (3-6 units/day) | 1.00 | 0.98 | - | 1.01 |
|  | Heavy drinker (7+ units/day) | 1.03 | 1.01 | - | 1.06 |
| Indices of multiple deprivation  (ref IMD 1) | IMD 2 | 1.00 | 0.99 | - | 1.01 |
|  | IMD 3 | 1.00 | 0.99 | - | 1.01 |
|  | IMD 4 | 1.02 | 1.01 | - | 1.03 |
|  | IMD 5 | 1.02 | 1.01 | - | 1.03 |
| Body Mass Index  (ref low weight) | Normal weight | 1.22 | 1.17 | - | 1.26 |
|  | Overweight | 1.51 | 1.45 | - | 1.56 |
|  | Obese | 1.90 | 1.83 | - | 1.97 |
|  | Very obese | 2.11 | 2.02 | - | 2.20 |
| Blood pressure | SBP (140-159; ref <130) | 1.62 | 1.61 | - | 1.64 |
|  | SBP (160-169; ref <130) | 3.85 | 3.81 | - | 3.89 |
|  | DBP (80-89; ref <80) | 1.02 | 1.01 | - | 1.03 |
|  | DBP (90-149; ref <80) | 2.39 | 2.36 | - | 2.41 |
| Cholesterol | Total (<6.2 vs >6.2 mmol/L) | 0.74 | 0.73 | - | 0.74 |
|  | HDL (<1.5 vs >1.5 mmol/L) | 1.00 | 0.99 | - | 1.01 |
| Cardiovascular risk | QRisk2 score (<10% vs >10%) | 4.08 | 4.04 | - | 4.12 |
| Frailty  (ref Fit) | Mild frailty | 1.61 | 1.59 | - | 1.63 |
|  | Moderate frailty | 1.01 | 0.98 | - | 1.04 |
|  | Severe frailty | 0.57 | 0.53 | - | 0.62 |
| Past medical history | Stroke | 0.98 | 0.96 | - | 1.00 |
|  | Myocardial infarction | 2.11 | 2.06 | - | 2.16 |
|  | Heart failure | 2.38 | 2.31 | - | 2.45 |
|  | Transient ischemic attack | 0.86 | 0.83 | - | 0.89 |
|  | Peripheral vascular disease | 0.81 | 0.78 | - | 0.84 |
|  | Angina | 2.02 | 1.98 | - | 2.07 |
|  | Coronary artery bypass graft | 1.45 | 1.38 | - | 1.52 |
|  | Chronic kidney disease | 2.18 | 2.13 | - | 2.22 |
|  | Diabetes | 1.05 | 1.04 | - | 1.07 |
|  | Atrial fibrillation | 1.46 | 1.43 | - | 1.49 |
|  | Cancer | 1.06 | 1.05 | - | 1.08 |
| Prescribed medications | Statins | 3.54 | 3.51 | - | 3.58 |
|  | Anti-thrombotics | 2.36 | 2.33 | - | 2.38 |
|  | Anticholinergics | 0.81 | 0.80 | - | 0.82 |
|  | Antidepressants | 1.09 | 1.08 | - | 1.10 |
|  | Hypnotics/anxiolytics | 0.93 | 0.92 | - | 0.94 |
|  | Opioids | 0.88 | 0.87 | - | 0.89 |
| Other | Database (Aurum vs GOLD) | 0.93 | 0.92 | - | 0.94 |

IMD = Indices of multiple deprivation; SBP = Systolic blood pressure; DBP = Diastolic blood pressure; HDL = High-Density Lipoprotein; CI = Confidence interval
