# Supplementary material for: The association between antihypertensive treatment and serious adverse events by age and frailty: A cohort study
Source: PLoS Med. 2023 Apr 19;20(4):e1004223. doi: 10.1371/journal.pmed.1004223 (PMC10155987; doi:10.1371/journal.pmed.1004223)
Supplement: S6 Table — *Proportions based on the number of patients with data available (i.e., excluding those with missing values). †IMD, indices of multiple deprivation; IMD score of 5 indicates patients in the highest quintile of deprivation (most deprived). (DOCX) [file pmed.1004223.s011.docx]

**S6 Table.** Baseline characteristics of the propensity score matched cohort

| Characteristic | No antihypertensive prescription during 12 month exposure period (non-exposed) | | Antihypertensive prescription during 12 month exposure period (exposed) | | \| **Standardised mean difference between treated vs untreated** \| \| --- \| | |
| --- | --- | --- | --- | --- | --- | --- | --- |
|  | **Mean/number** | **SD/%** | **Mean/number** | **SD/%** | **Pre-PS matching** | **Post-PS matching** |
| Total population | 429,800 |  | 429,800 |  | 3,834,056 | 859,600 |
| Age (years) (SD) | 62.5 | 13.0 | 61.3 | 13.0 | 0.49 | -0.09 |
| Sex (% female) | 212,321 | 49.4% | 215,140 | 50.1% | -0.02 | -0.01 |
| White ethnicity (%)* | 231,214 | 73.8% | 235,484 | 73.4% | 0.12 | -0.01 |
| Black ethnicity (%)* | 12,390 | 4.0% | 12,644 | 3.9% | 0.06 | 0.00 |
| South Asian ethnicity (%)* | 10,488 | 3.3% | 10,766 | 3.4% | 0.05 | 0.00 |
| Other ethnicity (%)* | 59,089 | 18.9% | 61,781 | 19.3% | -0.17 | 0.01 |
| High deprivation (IMD score of 5) (%)*† | 71,328 | 17.7% | 71,239 | 17.8% | 0.08 | 0.00 |
| Current smoking status (%)* | 87,285 | 21.9% | 88,504 | 21.7% | -0.07 | 0.00 |
| Alcohol consumption (heavy drinker) (%)* | 7,810 | 2.5% | 8,218 | 2.6% | 0.01 | 0.00 |
| Body mass index (kg/m2) (SD) | 28.2 | 5.6 | 28.2 | 5.6 | 0.28 | 0.01 |
| Systolic blood pressure (mmHg) (SD) | 149.4 | 13.1 | 150.1 | 13.6 | 0.87 | 0.06 |
| Diastolic blood pressure (mmHg) (SD) | 86.9 | 10.1 | 88.0 | 11.7 | 0.56 | 0.11 |
| QRisk2 risk score (SD) | 0.18 | 0.13 | 0.21 | 0.15 | 1.04 | 0.21 |
| eFrailty index score (SD) | 0.06 | 0.07 | 0.07 | 0.06 | 0.83 | 0.20 |
| Co-morbidities |  |  |  |  |  |  |
| Stroke (%) | 15,228 | 3.5% | 15,028 | 3.5% | 0.26 | 0.00 |
| Transient ischemic attack (%) | 7,388 | 1.7% | 7,253 | 1.7% | 0.18 | 0.00 |
| Myocardial infarction (%) | 12,345 | 2.9% | 11,919 | 2.8% | 0.65 | -0.01 |
| Heart failure (%) | 7,642 | 1.8% | 7,236 | 1.7% | 0.41 | -0.01 |
| Peripheral vascular disease (%) | 5,843 | 1.4% | 5,691 | 1.3% | 0.18 | 0.00 |
| Coronary artery bypass graft (%) | 2,641 | 0.6% | 2,501 | 0.6% | 0.37 | 0.00 |
| Angina (%) | 16,968 | 3.9% | 16,402 | 3.8% | 0.64 | -0.01 |
| Atrial fibrillation (%) | 17,329 | 4.0% | 16,964 | 3.9% | 0.39 | 0.00 |
| Diabetes (%) | 54,386 | 12.7% | 53,285 | 12.4% | 0.49 | -0.01 |
| Chronic kidney disease (%) | 13,372 | 3.1% | 12,924 | 3.0% | 0.39 | 0.00 |
| Cancer (%) | 20,777 | 4.8% | 21,130 | 4.9% | 0.08 | 0.00 |
| Treatment prescriptions |  |  |  |  |  |  |
| ACE inhibitors (%) | - | - | 156,794 | 36.5% | - | - |
| Angiotensin II receptor blockers (%) | - | - | 40,435 | 9.4% | - | - |
| Calcium channel blockers (%) | - | - | 121,910 | 28.4% | - | - |
| Thiazides and thiazide-like diuretics (%) | - | - | 136,447 | 31.7% | - | - |
| Beta-blockers (%) | - | - | 138,661 | 32.3% | - | - |
| Alpha-blockers (%) | - | - | 16,800 | 3.9% | - | - |
| Other antihypertensives (%) | - | - | 7,572 | 1.8% | - | - |
| Statins (%) | 102,357 | 23.8% | 102,978 | 24.0% | 1.02 | 0.00 |
| Anti-thrombotics (%) | 103,191 | 24.0% | 102,585 | 23.9% | 0.93 | 0.00 |
| Anticholinergics (%) | 36,922 | 8.6% | 36,493 | 8.5% | -0.02 | -0.01 |
| Antidepressants (%) | 80,610 | 18.8% | 78,986 | 18.4% | 0.01 | -0.01 |
| Hypnotics/anxiolytics (%) | 72,610 | 16.9% | 71,435 | 16.6% | -0.03 | -0.01 |
| Opioids (%) | 124,112 | 28.9% | 123,760 | 28.8% | 0.03 | 0.00 |

*Proportions based on the number of patients with data available (i.e. excluding those with missing values)

†IMD = indices of multiple deprivation; IMD score of 5 indicates patients in the highest quintile of deprivation (most deprived)
