# Supplementary material for: The association between antihypertensive treatment and serious adverse events by age and frailty: A cohort study
Source: PLoS Med. 2023 Apr 19;20(4):e1004223. doi: 10.1371/journal.pmed.1004223 (PMC10155987; doi:10.1371/journal.pmed.1004223)
Supplement: S7 Table — *Models adjusted for propensity score. ‡Number need to treat to prevent 1 event. (DOCX) [file pmed.1004223.s012.docx]

**S7 Table.** Post-hoc analyses of any serious adverse event and mortality

| **Outcome** | **Unadjusted analyses** | | **Adjusted analyses*** | | **Absolute risk difference (additional events per 10,000 patients per year)** | | **Number needed to harm/treat** | |
| --- | --- | --- | --- | --- | --- | --- | --- | --- |
|  | **Hazard ratio** | **95% CI** | **Hazard** **ratio** | **95% CI** | **Events** | **95% CI** | **5 years** | **10 years** |
| Any serious adverse event | 1.99 | 1.97 to 2.00 | 1.19 | 1.18 to 1.20 | 23 | 21 to 24 | 94 | 43 |
| All-cause mortality | 1.80 | 1.79 to 1.82 | 0.94 | 0.93 to 0.95 | -7 | -9 to -6 | 284‡ | 137‡ |

* Models adjusted for propensity score

‡ Number need to treat to *prevent* one event
