# Supplementary material for: The association between antihypertensive treatment and serious adverse events by age and frailty: A cohort study
Source: PLoS Med. 2023 Apr 19;20(4):e1004223. doi: 10.1371/journal.pmed.1004223 (PMC10155987; doi:10.1371/journal.pmed.1004223)
Supplement: S8 Table — *For the model accounting for competing risks, the sub-hazard ratio is presented. (DOCX) [file pmed.1004223.s013.docx]

**S8 Table.** Sensitivity analyses examining assumptions about missing data and competing risks (falls outcome)

| Analysis method | Approach to deal with missing data | Total population | No antihypertensive prescription during 12 month exposure period (non-exposed) | | Antihypertensive prescription during 12 month exposure period (exposed) | | Hazard ratio* | 95% CI |
| --- | --- | --- | --- | --- | --- | --- | --- | --- |
|  |  |  | **Population** | **Events** | **Population** | **Events** |  |  |
| Propensity score adjustment | Multiple imputation  (primary analysis) | 3,833,684 | 484,145 | 14,951 | 3,349,539 | 48,610 | 1.23 | 1.21 to 1.26 |
| Propensity score adjustment | Multiple imputation  (including smoking + IMD) | 3,833,684 | 484,145 | 14,951 | 3,349,539 | 48,610 | 1.23 | 1.20 to 1.26 |
| Propensity score adjustment | Multiple imputation  (missing indicator for smoking + IMD) | 3,833,684 | 484,145 | 14,951 | 3,349,539 | 48,610 | 1.23 | 1.20 to 1.26 |
| Fine-Gray competing risks model (adjusted for propensity score) | Multiple imputation  (primary analysis) | 3,833,684 | 484,145 | 14,951 | 3,349,539 | 48,610 | 1.27 | 1.24 to 1.30 |

*For the model accounting for competing risks, the sub-hazard ratio is presented
