# Supplementary material for: The association between antihypertensive treatment and serious adverse events by age and frailty: A cohort study
Source: PLoS Med. 2023 Apr 19;20(4):e1004223. doi: 10.1371/journal.pmed.1004223 (PMC10155987; doi:10.1371/journal.pmed.1004223)
Supplement: Attachment — Submitted filename: Response to reviewers 23.01.23.docx [file pmed.1004223.s016.docx]

**Response to reviewer comments**

**Requests from the editors:**

Please revise your title, restructure your abstract, add email addresses to the data availability statement and add a non-technical Author Summary to the manuscript. Please also update the referencing style and cite your supporting information appropriately.

These have all be added and/or updated accordingly.

Abstract Methods and Findings:

* In the last sentence of the Abstract Methods and Findings section, please describe the main limitation(s) of the study's methodology.

We have added the following sentence to the abstract:

“Although treatment effect estimates fell within the 95% CIs of those from randomised trials, these analyses were observational in nature and so bias from unmeasured confounding cannot be ruled out.”

Please confirm that the CPRD Independent Scientific Advisory Committee provided ethical approval for the study (or provide the name of the institutional review board that did).

We have added the following:

Page 4, line 132:

“The CPRD has global ethical approval for the use of anonymised electronic health records for research purposes, subject to approval of a study protocol by their Independent Scientific Advisory Committee. The protocol for this study was given prospective approval in February 2019 (ISAC protocol number 19_042) and is provided in the supplementary information (S15 Protocol).”

Please ensure that the study is reported according to the RECORD guideline, and include the completed RECORD checklist as Supporting Information. Please add the following statement, or similar, to the Methods: "This study is reported as per the REporting of studies Conducted using Observational Routinely-collected Data (RECORD) guideline (S1 Checklist)."

The checklist was provided previously and has been updated. It is now cited in the methods text as suggested.

Please state whether your study had a prospective protocol or analysis plan early in the Methods section. If a prospective analysis plan was used in designing the study, please include the relevant prospectively written document with your revised manuscript as a Supporting Information file to be published alongside your study, and cite it in the Methods section. A legend for this file should be included at the end of your manuscript.

We now include the protocol in the supporting information and cite it as follows:

Page 4, line 134:

“The protocol for this study was given prospective approval in February 2019 (ISAC protocol number 19_042) and is provided in the supplementary information (S15 Protocol).”

Changes in the analysis-- including those made in response to peer review comments-- should be identified as such in the Methods section of the paper, with rationale.

We have added the following:

Page 5, line 157:

“In response to peer review comments, all-cause mortality and a composite outcome of serious adverse events were examined as further post-hoc outcomes. Serious adverse events were defined as any hospitalisation or death with a primary diagnosis of the conditions mentioned above (with the exception of gout).”

Please provide the unadjusted comparisons as well as the adjusted comparisons in Table 2.

These have been added.

The asterisks in the legends of Figures 1-3 lack a corresponding flag in the Figures themselves.

These are simply footnotes to the figures, but do not relate to any specific aspect of each figure. These asterisks have now been removed.

Please present and organize the Discussion as follows: a short, clear summary of the article's findings; what the study adds to existing research and where and why the results may differ from previous research; strengths and limitations of the study; implications and next steps for research, clinical practice, and/or public policy; one-paragraph conclusion.

We have restructured as suggested.

Please temper claims of primacy of results by stating, "to our knowledge" or something similar.

We have amended as follows:

Page 8, line 292:

“These data confirm the association between antihypertensive treatment and serious adverse events[9], and *to our knowledge,* show for the first time how an individual’s absolute risk of harm changes with increasing age and frailty”

Page 9, line 325:

“*To our knowledge,* this was the largest population-based analysis of serious adverse events associated with antihypertensive therapy conducted to date[14], and the first to examine how these associations vary by age and frailty.

Please remove the ‘Conflicts of interest’, ‘Funding’, and ‘Data sharing statement’ sections from the manuscript – this information is captured via the Submission Form Questionnaire. Please delete the ‘Role of the funder/sponsor’ section.

These have been removed.

**Comments from the reviewers:**

**Reviewer #1: General remarks:**

This very large population-based, observational study importantly adds to current literature by detailing the probabilities of adverse effects in various age and frailty groups. Because RCTs usually include better-functioning patients, the results are apt to fill gaps in current knowledge. I do not have relevant criticisms about methods and analyses, which seem appropriate for the purpose. My main comments are for the discussion and interpretation of results.

1. When considering treatment of older patients, a fundamental question is whether the treatment is ongoing or started when they enter old age. The results promote caution when treating older and frail people with antihypertensives. However, as far as I understand, participants were included without previous treatment. Therefore, the results may give a wrong signal about treatment in older people who are already using the drugs when entering old age. Cardiovascular risk including hypertension is a risk factor of frailty, so the fact that hypertension treatment should be started for risk patients before old age and before they get frail. Deprescribing due to old age alone may also involve risks. Please discuss those points, eg. in Limitations.

We thank the reviewer for raising this good point. The focus on patients starting treatment was deliberate as it was thought this might lead to a more robust comparison of treatment effects, not confounded by prior antihypertensive use. However, this does raise the possibility that the findings may not extend to those who have already been prescribed treatment for many years. We have therefore removed our reference to deprescribing from the abstract and now discuss this limitation in the discussion section.

Page 9, line 352:

“Analyses focussed on new users of antihypertensive treatment to avoid the observed associations being confounded by previous prescription of antihypertensive treatment. However, this means that the results only reflect the association between antihypertensive treatment and serious adverse events in patients within 10 years of starting therapy. The association between treatment and serious adverse events in those already prescribed therapy for longer periods may be different.”

2. Line 65 " Antihypertensives are also the most commonly prescribed medications in patients admitted to hospital with adverse drug reactions". My comment: Maybe "AMONG the most commonly prescribed medications..." would be more appropriate. Often anticoagulants/NSAIDs/insulin/aspirin are holding the first place?

Thank you for this suggestion – we agree and have added ‘among’ to the sentence.

3. The extensive and useful review (Benetos A, et al.Circ Res. 2019 Mar 29;124(7):1045-1060 could be added to reference list, especially as it calls out for studies like yours.

We have added the following sentence:

Page 4, line 108:

“In addition, it was not possible to determine how treatment effects vary by patient level characteristics such as age or frailty, due to the absence of individual patient data. Such information is critical for clinicians, particularly when making individualised treatment decisions in these sub-populations. *Indeed, there is increasing support for the consideration of frailty status when prescribing of antihypertensive treatment[11], but in order to do this, better evidence is needed on the adverse effects of therapy in frailty subpopulations.”*

4. In RCTs like HYVET and SPRINT the authors have also considered frailty in relation to their results (Warwick J, et al. BMC Med. 2015 Apr 9;13:78). I agree that those trials did not include most frail patients, nevertheless, participants were old. I think it would be appropriate to acknowledge those substudies and their message

We agreed and now acknowledge these studies in the discussion:

Page 8, line 307:

“This was also reported in another review focussing exclusively on falls[23], although both studies included heterogeneous populations, and may have underrepresented patients with advancing age and frailty[24]. *Some trials focussing on older populations have presented results stratified by frailty, and concluded little association between treatment and serious adverse events[25, 26]. However, these studies typically recruited healthier older people and were therefore not representative of those with moderate to severe frailty living in the community[24].”*

**Reviewer #2:**

1. This paper addresses a very important topic that can, as investigators state, help inform clinical decision making about net benefits and harms of antihypertensive treatment. This is a particularly relevant issue given the recent move towards more aggressive blood pressure targets. The manuscript is well written and there are strengths in the design and population as I note. Because of the potential importance of results, I think it is important for investigators to address some important issues. Design The clinical population-based observational study is probably the optimal approach to determining adverse effects of antihypertensive treatment as clinical trial data has the inherent limitations of selection bias as well as relatively short follow up. Replicating the beneficial outcomes associated with antihypertensive treatment seen in clinical trials further enhances confidence in this observational study despite the inherent limitations of observational data.

We thank the reviewer for recognising the importance of this work and the strengths of our approach.

2. The study start date (1st January 1998) means that these data are now up 25 years old. Antihypertensive treatment regimens have changed; adverse effects of current antihypertensive regimens may differ from those studied. For example, investigators note that, "(63.6%) patients were prescribed one antihypertensive medication, 131,342 (27.1%) were prescribed two and 45,139 (9.3%) were prescribed three or more medications." at least in the United states, the prevalence of two or three antihypertensives are much higher than in this study. Furthermore, the classes of antihypertensive medications have likely changed. For example, a much smaller percentage of individuals in this court were on calcium channel blockers than today. this should at a minimum be addressed in the discussion section. Preferably, there would be a secondary analysis, limited to more recent data.

We thank the reviewer for raising this point. Although patients were able to enter the cohort on 1^st^ January 1998, less than 3% actually did, and more than 50% of the cohort entered the study in the past 15 years (see new S7 Figure). Some of the differences described above are likely to be due to differences in prescribing patterns in the UK compared to the US (for example, calcium channel blockers are not a first line therapy for hypertension until old age and combination therapies are rarely used in the UK). We are not convinced that further sensitivity analyses would address this limitation but we do agree that this needs to be acknowledged in the limitations and have now added new data showing when patients entered the cohort and the following additions to the text:

Page 6, Line 229:

“Patients entered the cohort throughout the period of observation (between 1998 and 2019, S7 Figure) and were followed up for a median of 7.0 years (interquartile range [IQR] 3.0 to 10.0 years).

Page 10, line 369:

“*In addition, the study start date was 1st January 1998 and more than 50% of the cohort entered the cohort in the last 15 years. Although antihypertensive prescribing trends may have changed during the whole study period, it is unclear whether this would affect the associations observed in the present study*.”

3. A strength of this study is the large and representative sample, although limited to a single country. Results will need to be replicated in other countries with different population characteristics and different antihypertensive treatment regimens.

We agree that there would be value in replicating these results in other settings. We have added the following to our discussion of the limitations of this work:

Page 10, line 371:

“*These prescribing trends reflect those in the UK during the study period and may have differed from those in other parts of the world. Ideally, these results should be replicated using data from other countries with different population characteristics and different antihypertensive treatment regimens*.”

4. The focus of the paper is on adverse events from antihypertensives. The population of significant clinical concern in which it is less clear whether the benefits outweigh the harms is the older population. There is little doubt that benefits outweigh harms in younger populations. Therefore, why was the population 40 and older rather than the clinically relevant population such as 60 or 65 and older? Similar to limiting systolic blood pressure to those <180, this study would be strengthened by limiting the population to those in whom there is question benefit outweighing harm. While secondary data by age are presented in the appendix, the primary results are diluted by the younger population.

We completely agree and think that our secondary analyses illustrate this point nicely. Inclusion of those aged 40 years and older was deliberate, since this is the age group in which antihypertensive treatment is typically first considered. Our analyses show the risk to be very low in this age group and we think these data are helpful and reassuring for prescribers. Inclusion of these patients also provides a helpful comparison for our subgroup analyses in older patients where the harms are notably more common. This analysis is presented in the main paper (not the appendix as suggested) and discussed in detail in the discussion section. We believe that these subgroup analyses already allow the reader to see how the risks change in the populations of interest and therefore have not made any further changes to the manuscript in response to this point.

5. Of note, a surprisingly small percentage of individuals with hypertension seem to have received antihypertensive treatment anytime during the 10 years of follow up. It appears that only 12% were on treatment at baseline and only 28% of those not on treatment initially received treatment during follow up, meaning only 40% were ever treated. This is a much smaller number than would be expected in the United States for example. Perhaps this may be related to the fact that they define hypertension as greater than 130 systolic. Treatment cut off of 140 systolic would have been clinical indicator for treatment during the time period of this study. therefore, systolic 140-180 appropriate range for this study

The inclusion of patients with a systolic of 130 mmHg or greater was chosen to reflect more recent guidance in the US. Of those with initial systolic blood pressure between 130-139 mmHg, 106,057 (6%) went on to be prescribed therapy during the exposure period. As suggested, this was lower than the proportion prescribed treatment with a systolic blood pressure between 140-180 mmHg (378,397 patients [21%]). However, this was not an analysis of prescription trends, but rather a comparison of serious adverse events in those exposed to treatment vs. those not exposed. Our analyses were adjusted for baseline blood pressure and so if this did have a modifying effect on the observed associations, this would have been accounted for in the analysis. We therefore think our chosen population is appropriate and have not made any changes to the manuscript.

6. "Exposure to antihypertensive medication was defined by the most recent prescriptions in the 12 months following cohort entry. The index date was defined at the end of this exposure period, after which patients were followed up for up to ten years. " Patients were defined as on or off treatment based on treatment status of the index date. obviously, there are going to be many changes in treatment over 10 years of follow up. Assuming treatment versus non treatment over 10 years based on one point in time, while perhaps matching "intention to treat" approach of randomized controlled trials does not provide confidence that adverse effects can be linked to antihypertensive treatment as participants may or may not been on treatment at the time. Analysis according to antihypertensive treatment exposure byintervals such as month would seem to be a better approach to the stated aims of this study. Investigators appear to have had access to antihypertensive prescriptions over the 10 years of follow up based on eTable 4 data which showed that 28% of "non exposed" participants received anti hypertensive during follow up. Investigators state in the discussion that their approach provides a conservative estimate of risk of adverse effects with antihypertensive treatment. They further note that more complicated analysis would be subject to other biases. While this is true, finding similar relationship between antihypertensive treatment and adverse outcomes with this additional analysis would provide greater confidence in the results.

We thank the reviewer for this suggestion but disagree that undertaking this further analysis would provide greater confidence in the results. As suggested in the manuscript, undertaking an analysis of time varying covariates, in combination with propensity scores and multiple imputation would require further statistical assumptions which are not well understood or described in the literature. The results of such an analysis may be subject to biases which are not fully understood. We note that statistical reviewer 3 agreed with this point of view and concluded that inclusion of further complex analyses would be too much and that our current approach is very robust.

Further, we note that our findings for most outcomes (including new analyses of all-cause mortality described below) were comparable with those from meta-analyses of randomised studies which are not subject to bias from confounding by indication. Therefore, our comparisons at least represent the effects of taking more antihypertensive treatment vs. less antihypertensive treatment and are likely to be a conservative estimate of the true underlying adverse event signal. Given that these data could be used to justify not starting treatment which may carry some benefit, we would suggest that such conservative estimates are appropriate. These arguments are detailed in the discussion:

Page 9, line 341:

“The present analyses used an intention to treat approach and did not account for those patients starting treatment in the control group (936,404 [28%]). This makes the findings comparable to randomised controlled trials, but potentially underestimates the true adverse event signal. More complex analyses are possible to address this issue, for example using time-varying covariates[30], however, the addition of such analyses to those already employed (multiple imputation with propensity scores) would require further statistical assumptions which are not well understood in the literature. Given that patients in the exposure group were typically exposed to treatment for much longer that those in the non-exposed group (median 0 years vs. 6 years), these treatment effect estimates should be viewed as conservative estimates of the true underlying adverse event signal. Given that these data could be used to justify not starting treatment which could carry some benefit, such conservative estimates are appropriate.”

7. The individual adverse effects included seem appropriate. However, they were each studied as separate outcomes. To better evaluate the overall adverse effects of antihypertensive treatment, a composite outcome of the serious adverse effects would be most appropriate from a clinical perspective. This would parallel the use of MACE cardiovascular outcomes. And many studies, a single outcome such as stroke does not reach statistical significant while the composite outcome does.

We did have sufficient power to study single endpoints due to our large sample size and previously chose not to examine a composite outcome due to the varying severity / impact of the outcomes studied. For example, a serious fall may have different long term consequences to someone experiencing electrolyte abnormalities or gout. However, we do agree that there is some value in a composite outcome comparable to MACE and have added this analysis to the paper (results described in S12 table). We report these results as follows, but have chosen to focus the discussion on our pre-specified single endpoints:

Page 5, line 157:

“*In response to peer review comments, all-cause mortality and a composite outcomes of serious adverse events were examined in further post hoc analyses. Serious adverse events were defined as first hospitalisation or death with a primary diagnosis of any of the conditions mentioned above (with the exception of gout which was not included because it is typically less serious and usually only captured in primary care records).”*

Page 7, line 259:

“*Post-hoc analyses confirmed that antihypertensive treatment is associated with an increased risk of any adverse event (examined as a composite outcome) and a reduced risk of all-cause mortality (S12 Table).*”

8. As the relevant clinical question is net benefit versus harm of antihypertensives, it would be helpful, at least as a secondary analysis, to see the relationship between antihypertensive treatment and all cause mortality, the relevant survival outcome for older adults, particularly those who are frail, who have multiple contributors to mortality.

We have added this analysis as suggested. The results show antihypertensive treatment to be associated with a small reduction in all-cause mortality, once again matching the findings of our previous meta-analyses of randomised controlled trials for adverse events (Albasri BMJ 2021; 372 doi: <https://doi.org/10.1136/bmj.n189>). We have added the following to the results describing this new analysis:

Page 7, line 259:

“*Post-hoc analyses confirmed that antihypertensive treatment is associated with an increased risk of any adverse event (examined as a composite outcome) but overall a reduced risk of all-cause mortality (S12 Table).”*

9. Summary: This paper addresses a very important topic and can, as investigators state, help inform clinical decision making. Given its importance, I would urge the investigators to do the analysis and present the data that can strengthen confidence and results. I would suggest limiting the analysis to the older population with systolic BP 140-180 for whom the question of benefit versus harm of antihypertensives is clinically relevant. Second, I would suggest doing the time varying analysis to see that the relationship between exposure and adverse effects holds up. Third, I think it would be helpful to have a composite measure of adverse effects to parallel MACE.

We have added the analyses of all-cause mortality and composite adverse events as suggested. We believe that our analyses already show the association between treatment and adverse events in older patients and do not believe limiting the population to those with a systolic blood pressure above 140 will provide sufficient added value to the results (since baseline blood pressure was adjusted for in the analysis). Given the potential limitations of the time varying analyses, and taking on board the recommendations of Reviewer 3, we have not included this as a further sensitivity analysis. We hope these additional analyses and justifications will be satisfactory.

**Reviewer #3:**

1. Sheppard and colleagues present the results of a very large retrospective cohort study, looking at the association between the use of antihypertensive medications, and the incidence of a variety of adverse outcomes. This review considers the statistical aspects of the paper. Overall, these are very good. The definition of the cohort, exposure, and outcomes, are very clear. Various methods of propensity score and covariate adjustments are applied. Cox proportional hazards models are used to assess associations, and a competing risks analysis is done as a sensitivity analysis. The results are generally presented very clearly. Both relative and absolute differences are reported. Whilst the paper recognizes that more complex analyses could have been done, I agree that this would be too much - there is plenty in the paper as it stands, and the "ITT-like" approach is very robust. I do have quite a few comments, though I believe many are to do with the way things are described or presented.

We thank the reviewer for recognising the thoroughness of our approach and hope that we have been able to provide greater clarity in our responses and edits described below.

2. The main thing that confused me was the derivation of the propensity scores. Line 130 states that "Propensity scores were generated for each outcome of interest…" It is not clear to me why multiple propensity scores are being used. To me, the propensity score is based on the predicted probability of being exposed, and for each outcome, the exposure is the same. Surely the propensity score has nothing to do with the outcomes? However, lines 177 to 180 make it sounds more like I would expect - talking about predicting the likelihood of treatment, though in the supplement, eTable 5 is titled "Propensity score model (primary outcome [falls])" - I do not see why the PS model relates to the primary outcome in particular. eTable 6 then reports O/E ratios and AUCs in relation to each outcome, even though the PS model is predicting treatment. And, if the PS models are specific to each outcome, it seems odd that the O/E ratios and AUCs should be virtually identical for all outcomes. Similarly, the panels of eFigure 3 are indistinguishable, to my eye, so it is not clear to me what they are showing.

We apologise for causing this misunderstanding. The propensity score model is the same for all outcomes since it predicts treatment, not the outcome of interest. However, because our dataset contained missing data, we had to build an imputation model to generate our propensity score. As is considered good practice, we included the outcome of interest (for the main analysis) in this imputation model, but not in the subsequent propensity score model. This meant that we needed to generate 8 different imputation models, and therefore 8 subsequent propensity score models included the same predictor variables but the way missing data were imputed for each was potentially subtly different. As noted, these subsequent propensity score models were almost identical and in our attempts to be transparent, we now recognise that their presentation by outcome is confusing rather than helpful. We have therefore updated the wording in our methods and simplified the presentation in the supplementary material to reflect that only one propensity score for treatment was developed.

Page 5, line 185:

“Propensity scores were generated using multivariable logistic regression. *Models included the covariates listed above, with continuous variables categorised to account for non-linear associations with the outcome (the use of splines/fractional polynomials was explored but led to model convergence issues).* Missing data were present for some covariates (see supplement for details) and these were dealt with using multiple imputation with chained equations (20 imputations)[16].”

3. I note that in eTable 5, the continuous variables (e.g. age, BMI, BP, Cholesterol) are categorised for PS modelling. Would it be better to treat these as continuous predictors?

We categorised the continuous variables to account for non-linear associations with treatment prescription (the outcome of the propensity score). We considered the use of splines/fractional polynomials but this led to model convergence issues. Categorisation of continuous variables has the potential for loss of predictive information, however, our propensity score model showed good performance and covariate balance was good in the matched analysis suggestion this modelling decision is unlikely to have affected our findings. We have updated our methods section to clarify this:

Page 5, line 185:

“*Models included the covariates listed above, with continuous variables categorised to account for non-linear associations with the outcome (the use of splines/fractional polynomials was explored but led to model convergence issues).*”

4. The index date is taken as 12 months after the first SBP recording of 130 or more, and the exposure as being on antihypertensive medication at the index date. The unexposed group are described in the Tables as "No antihypertensive prescription", but this is not entirely accurate, I think. Exposure requires a prescription for at least 30 days, within the last 30 days prior to the index date. The unexposed could then include people who took some medication between cohort entry and the index date. It might be good to show some summary statistics in this regard - how many people were there like this? How much medication did they take?

We thank the reviewer for highlighting this point and apologise that our description of the methods here was not entirely correct. Patients in the exposure group were those who were prescribed at least one antihypertensive during the 12 month exposure window. Prescriptions listed in the baseline characteristics were those prescribed at the most recent time point prior to the index date. For most patients this was a prescription in the last 30 days. No one in the unexposed group were prescribed an antihypertensive during the 12 month exposure window. We have clarified this in the text, and modified our description of the groups in each table:

Page 5, line 164:

*“The main exposure was prescription of any antihypertensive medication as defined in the British National Formulary (see S4 Table for details)[14]. Patients were allocated to the exposure group if they were prescribed at least one antihypertensive medication during the 12 month exposure window and medications at baseline defined by the most recent prescriptions prior to the index date. Those not exposed during this period were included in the non-exposed group.”*

Tables 1, S8 Table, S11 Table, S13 Table

| No antihypertensive prescription *during 12 month exposure period* (non-exposed) | Antihypertensive prescription *during 12 month exposure period* (exposed) |
| --- | --- |

5. The covariates for adjustment and derivation of propensity scores are defined using data up to the index date. Some of this data could therefore be after the point at which someone was put onto antihypertensive medication, which could happen at any time between cohort entry and the index date. Would it be safer to use data prior to cohort entry to define the covariates?

We agree that this is a potential limitation but since the exposure window was short, we would expect the covariates of interest to remain relatively stable and therefore do not think it would adversely affect our study results. We have added a sentence to the discussion flagging this potential limitation:

Page 9, line 356:

*“Further, baseline covariates were defined at the index date, rather than cohort entry, so it is possible that some covariates reflected the characteristics of patients after treatment was prescribed, although the impact of this is likely to have been small given the short exposure period.”*

6. One interpretation of the results could be that whether or not someone ends up in the exposed group is simply a measure of whether the initial SBP reading of 130 or more was a true or a false positive. For many of those in the unexposed group, that first raised blood pressure might have been an isolated result. In comparison, those meeting the definition of exposure might represent a truly hypertensive group. Alternatively, some in the unexposed group might have been stimulated by their initial SBP reading to adopt a healthier lifestyle, and got their BP back under control without the need for medication, whereas the exposed group may have been more resistant to such efforts, so that the unexposed group are simply a healthier group in general. Even if the covariate and PS adjustment can account for these differences between the two groups, the differences in outcomes are not necessarily due to the exposure, since exposure (as defined in this study) could (at least in part) be a marker of true hypertension, or for the severity of whatever is causing the hypertension. I think the limitations section should say more about alternative explanations for the results.

We agree that in principle this is possible, particularly if the outcomes of interest were related to blood pressure control or hypertension. However, this study focussed on adverse events from treatment, which are predominately independent from blood pressure. Hypertension itself would not necessarily increase the risk of these adverse events (conversely getting blood pressure under control through lifestyle modification would not necessarily reduce the risk of falls or syncope). We have clarified our approach in the limitations:

Page 9, line 356:

*“It is possible that those patients included in the exposure group were more likely to be truly hypertensive (hence receiving antihypertensive treatment) and therefore the observed differences in outcomes reflect this difference in hypertensive status rather than differences in treatment prescription. However, this study focussed on adverse events associated with treatment, which are predominately independent from blood pressure. Hypertension itself would not necessarily increase the risk of these adverse events and conversely getting blood pressure under control through lifestyle modification would not necessarily reduce the risk of falls, syncope or other adverse events of interest.”*

7. There is no mention of the proportionality of hazards in the Cox models. How was this checked, and was the assumption OK?

The proportionality assumption was checked for each model and was not violated on any occasion. We have clarified this in the methods.

Page 6, line 203:

*“Model assumptions were checked through inspection of Schoenfeld residuals and survival curves for the main exposure.”*

8. The subgroup analyses are a good feature of the paper, showing how the risk/benefit balance shifts according to the underlying risk of the outcomes. The results state that there were no differences in relative associations between exposure and outcomes across subgroups, but looking at Figures 2 and 3, I am not totally convinced. Having some interaction p-values might help, but for me it definitely looks like there might be some trends in relative associations across age groups, in particular. The data must be available to test for interactions, either using categories of age, or with age as a continuous modifier of the associations.

We thank the reviewer for pointing this out and agree that on reflection, there are some differences in the relative effects, particularly for falls, acute kidney injury, electrolyte abnormalities and gout. We have refrained from adding p values for interaction since these can lead to spurious results, particularly when there a number of subgroup analyses as presented here. However we do now acknowledge these trends in our description of the results.

Page 7, line 264:

*“The relative association between antihypertensive treatment and serious adverse events increased with age for falls, acute kidney injury, electrolyte abnormalities and gout (figure 2). These trends were less obvious in subgroups by baseline frailty (figure 3). However, the estimated absolute risk of serious adverse events did increase substantially with both age and frailty, particularly for falls, acute kidney injury and electrolyte abnormalities (figures 2 and 3).”*
