# Supplementary material for: The association between antihypertensive treatment and serious adverse events by age and frailty: A cohort study
Source: PLoS Med. 2023 Apr 19;20(4):e1004223. doi: 10.1371/journal.pmed.1004223 (PMC10155987; doi:10.1371/journal.pmed.1004223)
Supplement: Attachment — Submitted filename: Requests from Editors.docx [file pmed.1004223.s017.docx]

Requests from Editors:

Abstract -- methods: Please briefly state which variables enter the propensity score.

Abstract – methods and findings: Before the sentence “Although treatment effect estimates fell within the 95% CIs of those from randomised trials, these analyses were observational in nature and so bias from unmeasured confounding cannot be ruled out.” it would be good to strongly emphasize that this data is superior to data from trials in that it provides a view of real-life effectiveness – the trial is unlikely to be externally valid, because trial participants are typically highly selected and diligently supported by the trial team in their treatment retention and adherence.

It would be good to also emphasize this strength of the present study in the “Why was this study done?” section in the Author Summary.

Abstract – methods and findings: Finally, it would also be good to mention that the results remained essentially the same when the competing risk of death from all causes was taken into account in sensitivity analyses.

Response: We have added all of the suggestions to the abstract of the paper and author summary
